# Atomic resolution protein allostery from the multi-state structure of a PDZ domain

**DOI:** 10.1038/s41467-022-33687-x

**Published:** 2022-10-20

**Authors:** Dzmitry Ashkinadze, Harindranath Kadavath, Aditya Pokharna, Celestine N. Chi, Michael Friedmann, Dean Strotz, Pratibha Kumari, Martina Minges, Riccardo Cadalbert, Stefan Königl, Peter Güntert, Beat Vögeli, Roland Riek

**Affiliations:** 1 Laboratory of Physical Chemistry, ETH Zürich, Vladimir-Prelog-Weg 2, CH-8093 Zürich, Switzerland; 2 Department of Medical Biochemistry and Microbiology, Uppsala University, Husargatan 3, 75121 Uppsala, Sweden; 3 Institute of Biophysical Chemistry, Center for Biomolecular Magnetic Resonance, Goethe University Frankfurt am Main, Frankfurt am Main, Germany; 4 Department of Chemistry, Tokyo Metropolitan University, Hachioji, Tokyo 1920397 Japan; 5 Biochemistry and Molecular Genetics Department, University of Colorado School of Medicine, Colorado, CO USA

**Keywords:** Solution-state NMR, Computational biophysics, Mechanism of action

## Abstract

Recent methodological advances in solution NMR allow the determination of multi-state protein structures and provide insights into structurally and dynamically correlated protein sites at atomic resolution. This is demonstrated in the present work for the well-studied PDZ2 domain of protein human tyrosine phosphatase 1E for which protein allostery had been predicted. Two-state protein structures were calculated for both the free form and in complex with the RA-GEF2 peptide using the exact nuclear Overhauser effect (eNOE) method. In the apo protein, an allosteric conformational selection step comprising almost 60% of the domain was detected with an “open” ligand welcoming state and a “closed” state that obstructs the binding site by changing the distance between the β-sheet 2, α-helix 2, and sidechains of residues Lys38 and Lys72. The observed induced fit-type apo-holo structural rearrangements are in line with the previously published evolution-based analysis covering ~25% of the domain with only a partial overlap with the protein allostery of the open form. These presented structural studies highlight the presence of a dedicated highly optimized and complex dynamic interplay of the PDZ2 domain owed by the structure-dynamics landscape.

## Abstract (web summary)

In this manuscript the authors report accurate multi-state protein structures of the PDZ domain using biological NMR. By looking into protein structural states, the authors report an allosteric pathway at atomic resolution that validates previously reported low resolution findings and uncovered a structural hallmark of the allosteric ligand binding to the PDZ domain.

## Introduction

An important area of structural biology is the elucidation of the molecular mechanisms behind enzyme activity, protein target recognition, and transduction of biological signals enabling various cellular pathways, that rely on protein fold and dynamics^1^. Correlated protein motion spanning across the scaffold is of particular interest as it gives rise to protein states and equilibrium^2,3^. One of the most studied conserved protein motions is ligand-induced protein rearrangement that enables to transduce the signal through a biomolecule in a specific manner known as allostery. There are two broadly accepted allosteric models including the population-shift model and the dynamic allostery model^4,5^. The population-shift model explains protein motion based on the protein population equilibrium between distinct protein conformations, whereas dynamic allostery relies on statistical thermodynamics that can explain dynamics-based signal transmission without a change in the average structure.

Typically for large systems including enzymes or multimeric proteins, allostery is structurally studied indirectly using X-ray crystallography of distinct states^6^. Despite the lack of atomic resolution, chemical shift or relaxation-based NMR studies were able to show allosteric communication within a protein fold in a solution under near-physiological conditions^7–13^. The ligand binding-induced chemical shift changes (for example, measured in a [^15^N,^1^H]-correlation experiment) can be used to probe both local direct ligand-induced protein conformational changes and changes far away from the ligand binding site, which are often attributed to allosteric interactions^14^. As chemical shifts are more sensitive to the direct interaction with the binding partner, shifts in the protein binding site usually dominate the allosterically invoked shifts.

The recent advances in biological NMR provide insights into the correlated protein sites at atomic resolution by determining multiple protein states using a plethora of NMR-based experimental restraints such as residual dipolar couplings (RDC), cross-correlated relaxation (CCR), paramagnetic relaxation enhancement (PRE), and Nuclear Overhauser Effect (NOE)^15–23^. In particular, the exact Nuclear Overhauser Effect (eNOE) method implemented in the eNORA2 package within CYANA yields experimental distances with a resolution of up to 0.1 Å^24–28^, enables the structure calculation of multiple protein states, and allows for correction of spin diffusion^24,25,29,30^. Furthermore, correlated protein sites can be studied using the distance and angle statistics of calculated protein conformers^31–34^.

One of the most studied families of allosteric molecules is PDZ domains^35^. These domains are crucial for protein-protein recognition and protein complex assemblies in multicellular organisms^36^. PDZ domains generally recognize the carboxyl-terminus of various target proteins and take part in many cellular processes including cell growth and proliferation. PDZ domains display a characteristic compact fold composed of six β-strands, two α-helices, and a unique flexible loop near the binding pocket^37^. The strands of the protein form an antiparallel β-sheet that serves as a platform for the binding of target molecules. Human tyrosine phosphatase 1E protein (hPTP1E) contains a PDZ2 domain and mediates a series of crucial biological processes such as protein-protein interaction^38,39^, signaling^40^, and apoptosis^41^. The C-terminal peptide derived from the Ras-associated guanine nucleotide exchange factor 2 (RA-GEF2) is a versatile binding partner of the PDZ2 domain of hPTP1E^38^. X-ray crystallography and solution NMR structures of the PDZ2 domain were solved for the free form as well as for the complex bound to the RA-GEF2 peptide^42–44^. The PDZ2 domain of hPTP1E binds RA-GEF2 by a β-strand addition between β-strand 2 and α-helix 2, similar to other PDZ domains^45^. The PDZ2 allostery was studied with various techniques including molecular dynamics^46,47^, evolutionary data^48^, and NMR relaxation of protein methyls^7^.

In this study, we showcase the use of the recently emerged eNOE approach to investigate multi-state structures showing correlated protein sites that may be used to infer an induced-fit ligand binding mode, and protein allostery enabled by the structural information of individual protein states at atomic resolution of both free and bound forms of the PDZ2 domain of hPTP1E.

## Results and discussion

### Ligand-induced dynamic changes of the PDZ2 domain

**Fig. 1:**
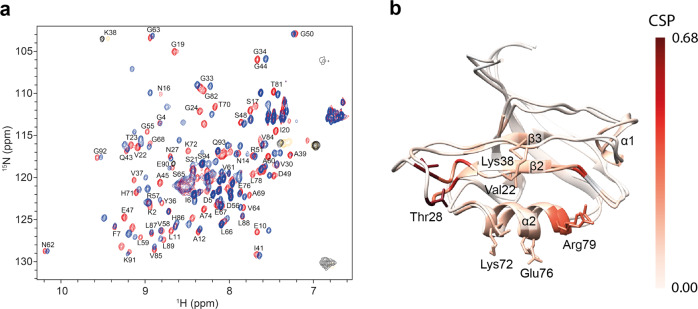
Ligand-binding induced conformational changes measured by chemical shifts. [^1^H,^15^N]-HSQC spectra for the PDZ2 domain in apo form (red) and bound to the RA-GEF2 peptide (blue) (**a**). Residues of the two-state PDZ2 apo structure with the lowest CYANA target function were colored according to the mean of the chemical shift perturbation of ^15^N and ^1^H (CSPs) as indicated by the bar on the right (**b**).

Heteronuclear 2D NMR spectroscopy was applied to gain a qualitative understanding of the binding of the PDZ2 domain of hPTP1E to the C-terminal, eight residue-long, peptide derived from the Ras-associated guanine nucleotide exchange factor 2 (RA-GEF2; Ac-ENEQVSAV-COOH) and allosteric interactions. A [^1^H,^15^N]-HSQC spectrum was acquired at 298 K for a uniformly ^15^N-enriched PDZ2 domain both for a free form and bound to the peptide supplied in a twofold excess. An overlay of the [^1^H,^15^N]-HSQC spectra together with the chemical shift perturbation (CSP) mapped on the protein 3D structure is shown in Fig. 1. Averaged atom-weighted chemical shift perturbations were mapped on the later calculated apo protein structure to delineate the allosteric system of the PDZ2 domain. Absolute CSP values are shown in Supplementary Fig. 1. In line with previous reports, residues of the PDZ2 binding site showed significant CSPs. Several residues far apart from the binding site also show CSP, indicating a highly dynamic scaffold^7,37^. Additionally, significant CSPs are observed for residues at the β-strand 2 and α-helix 2, which sandwich the ligand upon binding, with less prominent CSPs in the flexible loop Gly24-Gly34, β-strand 3, and α-helix 1. The β-strand 3 and α-helix 1 are far away from the binding site and thus have been identified as allosteric sites^7^.

### Multi-state structure determination of the PDZ2 domain

For apo PDZ2, 1553 eNOE-derived distance restraints were extracted from a set of 3D [^15^N,^13^C]-resolved [^1^H,^1^H]-NOESY-HSQC spectra recorded at mixing times of 8, 16, 24, 32, 40, 48, and 80 ms. These distance restraints comprised 410 bidirectional distance restraints of the highest precision. In addition, 130 scalar couplings were collected, which resulted in ~17 restraints per residue, as summarized in Supplementary Table 1. Similarly, for the complex PDZ2, 1484 distance restraints from eNOEs and 65 scalar couplings that resulted in ~16 restraints per residue were collected, as summarized in Supplementary Table 2.

With the given experimental input, multi-state structure calculation with the program CYANA can be performed. CYANA simultaneously optimizes multiple structural states by minimization of the target function (TF). TF is calculated by comparing the simulated distances, which are back-calculated from all optimized states, to the experimental upper and lower limit restraints extracted from the NOE cross-peaks. Individual states are kept in proximity of each other by weak restraints, but local movements with an amplitude below 1.2 Å are allowed without penalty. These restraints are termed symmetry restraints within CYANA because they have been previously used in symmetric oligomeric protein entities and are here applied to the two states correspondingly. Spin diffusion is a major factor causing inaccuracy in deriving distances from the NOEs^49^. Theoretically, spin diffusion can be accounted for if all NOE cross and diagonal peaks can be measured unambiguously, which is unrealistic given a limit to NMR sensitivity^50^. Therefore, an alternative version of the spin diffusion correction referred to as exact NOE by Relaxation matrix Analysis (eNORA) was implemented as a part of the eNOE technique that relies on a given 3D protein structure^24^. So far, eNOE analysis has successfully been applied to the proteins WW domain of Pin1, full-length Pin1, GB3, cyclophilin A, ubiquitin, and a small 14-mer UUCG RNA tetraloop^29,30,51–54^. The elucidation of the correlated states was recently automated with the software package PDBcor, which elucidates correlated protein sites in the form of structural correlations in an unbiased and automated way from the distance statistics of individual structural entities in a multi-state structure^32^. It can quantify correlations in structural ensembles, uncover the protein regions that undergo synchronized rearrangements, and optimally separate conformers into states. PDBcor uses information theory and systematic clustering of protein conformers to extract mutual information between individual residues^32^.

In the case of the PDZ2 domain, both apo and holo multi-state protein structures were calculated following the established protocol introduced above^29,51,52^ using eNORA2 for the spin diffusion correction and distance extraction^24,25^ and CYANA for the protein structure calculation with minor modifications^19^. Symmetry restraints that keep structural entities in proximity of each other were relaxed in the region starting from Gly24 up to Gly34 to allow for the additional motion amplitude of the PDZ2 flexible loop. The enhanced flexibility in the loop region was shown with ^15^N R_2_/R_1_ NMR relaxation rates (Supplementary Fig. 2). The structure annealing algorithm was executed with 150’000 energy minimization steps for 1000 two-state conformers. A series of 1–9 state structure calculations were performed (Supplementary Fig. 3), indicating that a single-state structure does not fulfill the experimental data well, as evidenced by the high CYANA TF, which is a measure of restraint violations. However, two states appear to be sufficient to describe the experimental data well (Supplementary Figs. 3, 4, 5, 8). The relative population of the two states could be estimated with the use of the CYANA TF to be about 1:1 (Supplementary Fig. 6). A jackknife procedure, in which a certain percentage of restraints is removed randomly, followed by two-state structure calculations and correlated states analysis with the PDBcor software demonstrates that the system is well determined. About 80% of the experimental data are required for the emergence of structural correlations (Supplementary Fig. 11). The multi-state nature of the PDZ2 domain was further validated by monitoring the local sidechain dynamics, specifically rotamer averaging of the methyl groups of valines and leucines, presence of single aromatic protons HD1/HD2 and HE1/HE2 for Phe7 and Tyr36, and scalar couplings requesting rotamer averaging (Supplementary Fig. 12). The multi-state nature of the PDZ2 domain is further requested from the ^15^N-relaxation data showing widespread dynamics (Supplementary Figs. 12, 13).

The 20 two-state conformers with the lowest TF were selected to represent the calculated two-state structure. They satisfy the experimental restraints well, as indicated by the low CYANA target function (see Supplementary Tables 1, 2) and show favorable Ramachandran plot statistics with less than 1% of the residues in the disallowed regions (Supplementary Table 3 and Supplementary Fig. 7). In addition, the resulting structures reproduce the known PDZ2 protein fold with root mean square deviations of 1.11 Å for apo and 1.34 Å for the complex from the corresponding reported crystal structures (Supplementary Tables 1,2).

### The two-state structures of the PDZ2 domain of the apo and holo forms

**Fig. 2:**
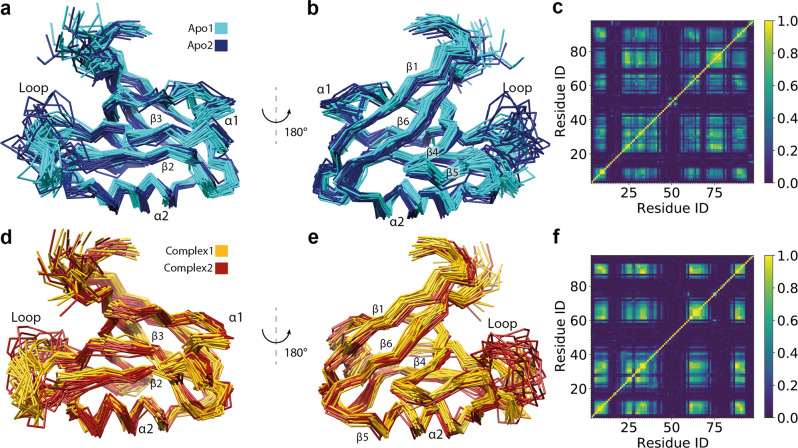
Presentation and correlation analysis of the calculated eNOE PDZ structures. Two-state ensemble structures of the PDZ2 domain in the ligand-free apo form (**a**, **b**) and bound to the RA-GEF2 peptide (holo) (**d**, **e**) calculated with eNORA2^24,25^ and CYANA software are shown in two different orientations. The two apo states are colored in cyan and dark blue, whereas the two holo states are colored in yellow and red. The secondary structures and the loop comprising residues Gly24-Gly34 are indicated. Structural correlations and optimal state separations for both protein ensembles were calculated with PDBcor in standard settings^32^ and shown as distance correlation matrix heatmaps for the apo (**c**) and holo (**f**) forms of the PDZ2 domain (0 (blue) means lack of any correlation and 1 (yellow) means 100% correlation between the two states in the bundle of conformers).

**Fig. 3:**
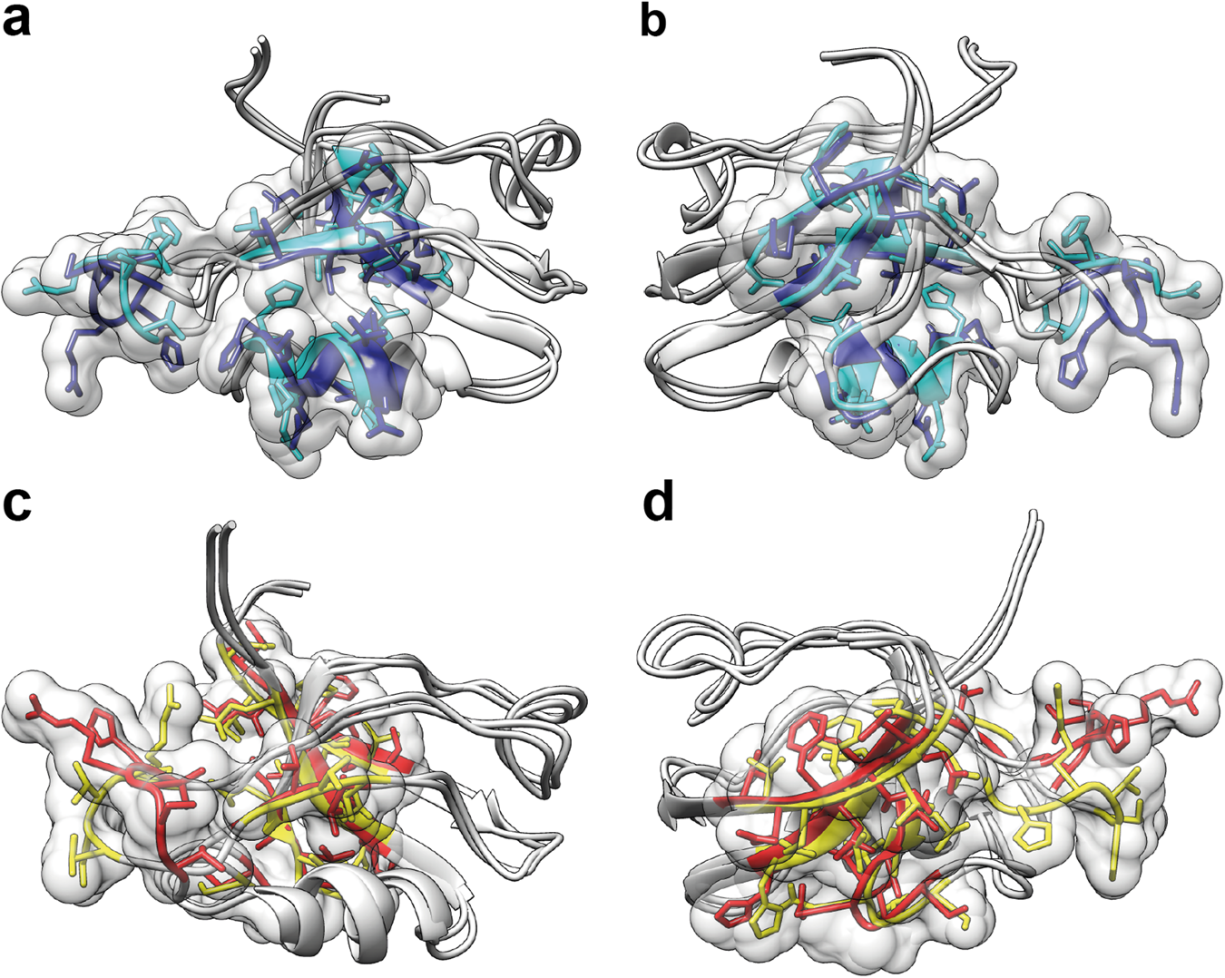
Localization of the residues responsible for the correlations in apo and holo forms of the PDZ domain. Core residues responsible for the two-state correlations of the apo state (**a**, **b**) are localized around the ligand-binding site and the protein’s hydrophobic core, whereas core residues responsible for the two-state correlations of the PDZ2 RA-GEF2 complex (**c**, **d**) are localized to the other side of the protein β-sheet. The 15 highest correlated residues, as determined by the correlation matrices shown in Fig. 2c, f are highlighted as a single volume entity on the two-state PDZ2 structures including state-dependent ribbon coloring and sidechain representation. As it is visible from the protein 3D structure, the highest correlations are concentrated in the protein binding site and all sites involved in the conformational selection mechanism shown in Figs. 4, 5.

The eNOE-based two-state structures of the free PDZ2 domain (apo) and in complex with the RA-GEF2 peptide (holo) represented by 20 conformers for each state are shown in Fig. 2a, b, d, e. The two-state structures were analyzed with the PDBcor software in standard settings^32^ generating heatmaps (Fig. 2c, f), which were used to color the structural states in cyan/blue for the apo and yellow/red for the holo PDZ2. Each state is represented by conformers color-coded accordingly (note that for convenience, the conformer separation and coloring will be kept consistent throughout the manuscript). Inspection of the apo two-state structure yields two separated states for the β-sheet, α-helix 2, and the flexible loop Gly24-Gly34. The flexible loop adopts two very distinct conformations, one of which is relatively loose and further apart from the binding site (a dark blue state in Fig. 2a, b), while α-helix 2 is shifted along the helix axis and β-strands 2 and 3 vertically to the β-strand arrow. Hence almost 60% of the entire protein domain appears to shuffle between two states with the aforementioned secondary structures around the peptide ligand-binding site comprising β-strand 2, α-helix 2, and the loop spanning residues Gly24-Gly34 but to a smaller degree extending also into the entire β-sheet. Core residues responsible for the two-state correlations of the apo state are localized around the ligand-binding site (see also below) and the protein’s hydrophobic core (Fig. 3a, b). In Fig. 3, the 15 highest correlated residues, as determined by the correlation matrix, are highlighted as a single volume entity on the two-state apo PDZ2 structure.

Since dynamics in loops is believed to be inherent, one may hypothesize that the dynamics of the two states may originate from the loop comprising residues Gly24-Gly34. Due to its partial flexibility enabled by the two double glycine hinge motifs Gly23-Gly24 and Gly33-Gly34 and its location at the edge of the protein structure, the loop harbors thermal intrinsic local dynamics. Because of the conformational space that the loop covers, the relative position of helix 2 is altered between the two states via the sidechains of Thr70 and His71 and vice versa, which means that the two segments are coupled conformationally. In addition, in state 1, the covered conformational space of the C-terminal end of the loop together with Ile35 is coupled with a shift of the β-sheet via Val58/Leu59 and vice versa.

In addition to the two correlated states, the local distinct configurations of the sidechains, indicated by the Chi1 angle values shown in Supplementary Figs. 4,5, are further supported by scalar coupling measurements, shown in Supplementary Fig. 8, and state-dependent Ramachandran plots of the backbone, shown in Supplementary Figs. 9, 10.

Interestingly, the holo two-state structure also yields two separated states, including the β-sheet 2 and the flexible loop Gly24-Gly34, similar to the apo structure, but not α-helix 2 (residues 71–80) (Figs. 2c, d, 3c, d). As for the apo form, the local distinct configurations are also indicated (Supplementary Fig. 4).

### Evidence for conformational selection in PDZ2 in terms of ligand binding

**Fig. 4:**
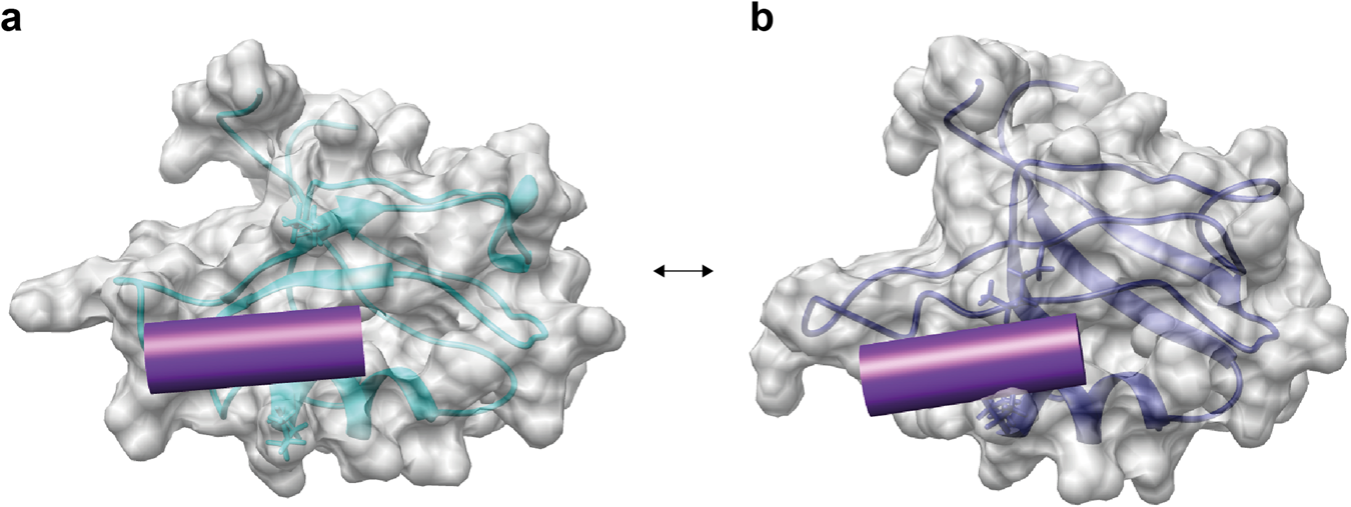
Illustration of the conformational selection mechanism in apo form of the PDZ domain. The apo form comprises an open ligand-welcoming (**a**) and a closed ligand-obstructing state (**b**). The surface views of the two states of the PDZ2 of the apo form, with state 1 (**a**) and state 2 (**b**) are shown with a transparent ribbon representation and the important sidechains of Lys38 and Lys72 (as sticks). The position of the RA-GEF2 peptide is visualized with a violet cylinder. The access to the binding groove is obstructed in PDZ2 apo state 2 by sidechains of residues Lys38 and Lys72 (**b**), which indicates the presence of a conformational selection mechanism upon ligand binding.

**Fig. 5:**
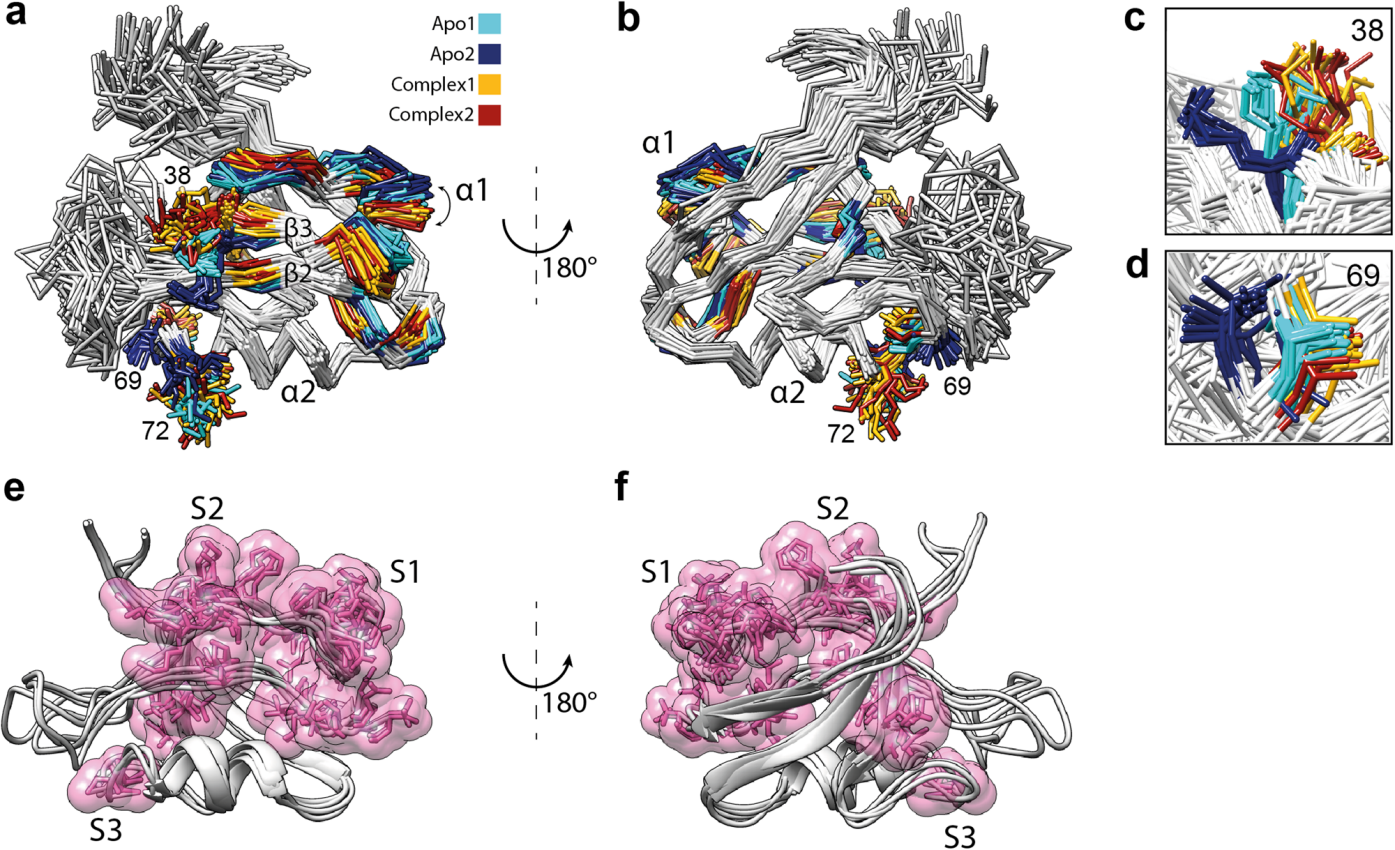
Ligand binding-induced structural changes of the PDZ2 domain. **a**, **b** Two views of the aligned apo and holo PDZ2 structural ensembles. All residues with distances between apo and holo averaged C_α_ positions of more than 1.5 Å except flexible regions are highlighted on the 3D protein structure with the color coding according to Fig. 2 with cyan and blue representing the apo form and yellow and red the holo form, respectively. Ligand-induced allosteric movement of the α-helix 1 is indicated by the arrow. Significant rearrangements in the α-helix 1 and part of the β-strand 2 facing it, the middle part of the β-sheet, and parts of the β-strand 5 and α-helix 2 in the proximity of the flexible loop validate previously reported allosteric interactions in the PDZ2 domain^7,48^. The conformational selection mechanism is indicated by sidechains of residues Lys38 (**c**), Ala69 (**d**), and Lys72 of the apo and holo structures. The sidechain superposition of apo state 1 (cyan) with both holo states (yellow and red) suggests a conformational selection mechanism on PDZ2 for ligand binding. **e**, **f** The three sites S1–S3 of the aforementioned correlated network. The elucidated correlated network spans from the binding site to the α-helix 1 through S1, to the Lys54 through S2, or to the Val58 through S3.

The presence of two states in the apo PDZ2 hints toward the presence of a conformational selection mechanism of ligand binding, since the binding pocket, comprised of α-helix 2 and β-strands 2, and β-strand 3 are distinct between the two states. In state 2 (blue) of the apo PDZ2 domain, the sidechains of residues Lys38 and Lys72 point directly to the binding site (Figs. 4b, 5a, c) and obstruct the binding groove. The local difference between the two states is attributed to a distinct backbone configuration for both Lys38 (Fig. 5c, with its CHI1 angle being in *trans*) and Lys72 with its CHI1 angle either in a *trans* or in *gauche*+ configuration, in line with scalar coupling measurements (Supplementary Fig. 8). This finding suggests that state 2 has to be a “closed” conformation, obstructing ligand-binding whereas state 1 (cyan) is the open, ligand-welcoming state that superimposes with the holo states (Figs. 4a, 5a). Furthermore, a further detailed investigation of both the apo and holo two-state structures with a focus on the sidechains of residues Ala69, Thr70, Lys72, and Lys38 close to the binding site suggests in part a conformational selection mechanism as both holo states (while distinct) are close to or are overlapping with apo state 1 (Fig. 5a, c, d). Based on the *R*_ex_ contribution to the ^15^N-relaxation shown in Supplementary Figs. 12, 13 indicating the presence of conformational exchange dynamics in loop 2, Lys38, and Ala69 the dynamics between the two states is indicated to be in the high nanosecond to microsecond time range. Conclusively, this indicates the presence of a conformational selection model for ligand binding.

### Ligand-induced conformational rearrangement of the PDZ2 domain

A conformational selection model for binding does not exclude the presence of an induced-fit mechanism as there have been several studies in this regard, including both two-step processes or simultaneous conformational selection and induced-fit mechanisms^55–59^. A systematic study of the conformational changes between the apo and holo PDZ2 scaffolds may provide insights into the nature of the binding mechanism. Conformational changes were first quantified in terms of the average distance between the apo and holo PDZ2 structures. For that purpose, individual conformers from both apo and holo two-state ensembles were superimposed to the first conformer of the apo ensemble using the Kabsch algorithm in UCSF Chimera^60^, then the C_α_ atom coordinates of all conformers were extracted and averaged over all apo and holo conformers to obtain mean apo and mean holo structures. Next, the distance between the C_α_ atom coordinates of the averaged apo and holo PDZ2 conformations was calculated for each residue. The residues for which the C_α_ deviate more than 1.5 Å from each other were highlighted and mapped on the apo PDZ2 structure as shown in Fig. 5. The loop comprising residues Gly24-Gly34 and the N-terminal flexible segment were excluded from the analysis due to their elevated flexibility. The largest ligand-induced backbone conformational changes are concentrated in three sites. The first site (S1) includes α-helix 1 and part of the β-strand 2 facing it. The second site (S2) includes the middle part of the β-sheet. The third site (S3) includes parts of the β-strand 5 and α-helix 2 in the proximity of the flexible loop, as summarized in Fig. 3. Ligand binding yields a shift of the α-helix 1 and a part of the β-strand 2 facing it to accommodate the ligand with a shift of the middle part of the β-sheet away from the binding site quantifiable also by the distance between the C_α_ atoms of residues Gly23 and Lys72, which is 7.8 ± 0.6 Å in the apo state, but 8.95 ± 0.5 Å for the PDZ2 complex state. The aforementioned structural rearrangements are coupled to the ligand-binding site, as backbone rearrangements between apo and complex PDZ2 domain are ligand-induced. Those findings correlate with one of the major findings of Lockless and Ranganathan, who showed a statistical coupling between His71 and distal residues Ala46 and Ile52 that are part of the α-helix 1 and to the findings of Fuentes et al. that indicated a coupling between residue Ile20 of the binding site and residues Ala39 and Val40 of the β-strand β3^7,48^.

The distinct two-state structures between the apo and holo PDZ2 domain discussed indicate an induced-fit-type mechanism as previously reported by stopped-flow measurements^61^. In the context of multidomain proteins, the induced-fit allosteric mechanism of the PDZ2 domain may connect the PDZ2 binding site with an interdomain interface. Despite the scarce information about the exact location of the potential interdomain interface for the PDZ2 domain from hPTP1E, it is known that Cdc42 directly interacts with α-helix 1 from the Par-6 PDZ domain^62^ and a domain-domain interaction between the PDZ1 and PDZ2 occurs through a site located at the α-helix 1 / β-strand 1 region^63^. This indicates a potential involvement of the α-helix 1 in the PDZ interdomain interface, which also experiences the largest ligand-induced rearrangement according to the presented eNOE studies upon ligand binding, including a ligand-induced structural correlation with the β -sheet and the flexible loop Gly24-Gly34.

### A comparison of the proposed allosteric pathways of the PDZ2 domain

**Fig. 6:**
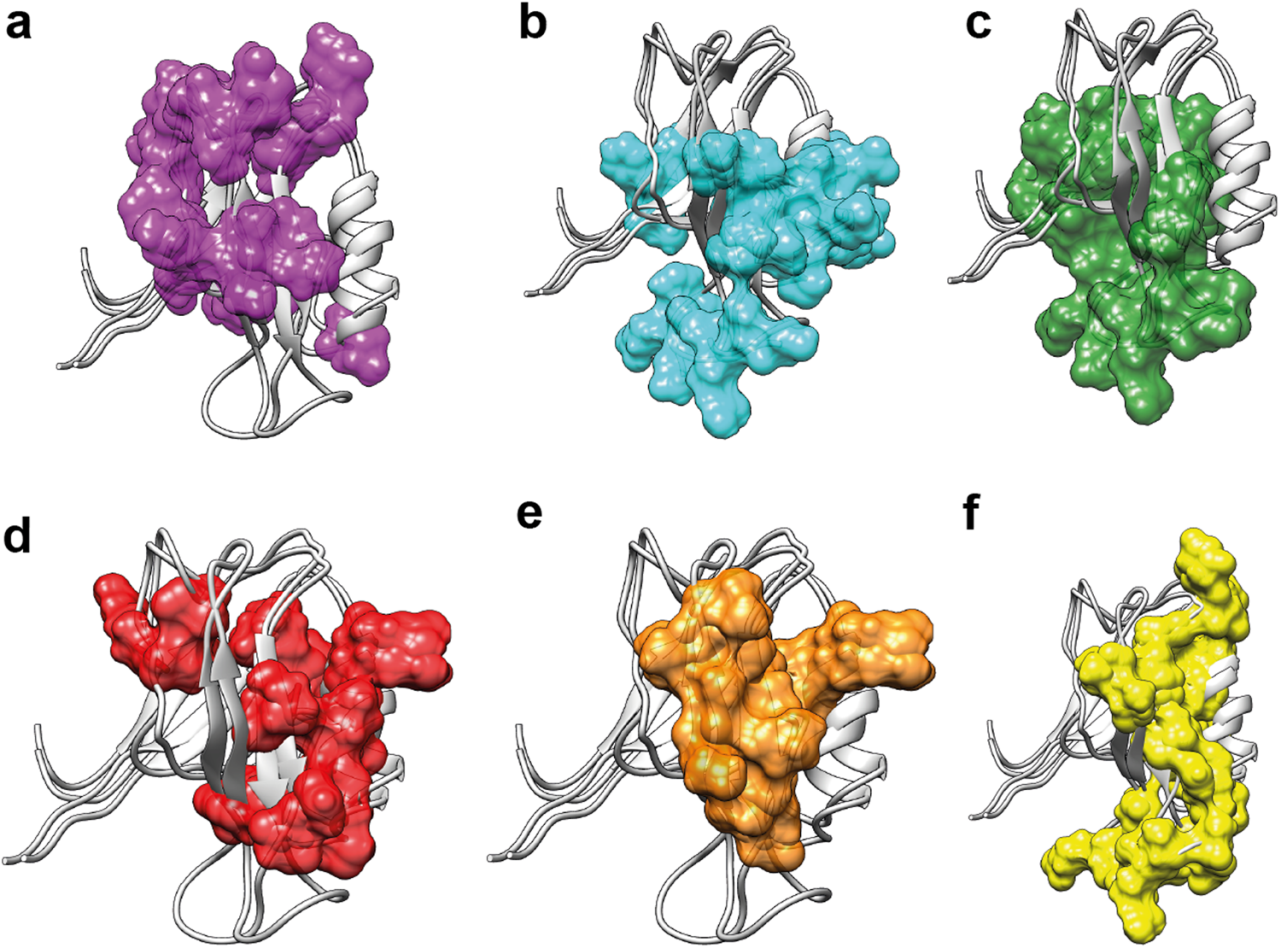
Summary of the PDZ allosteric networks from the literature and our study. Proposed allosteric pathways within the PDZ2 domain determined or predicted with various methods, including eNOE-based induced-fit allosteric network (**a**), eNOE-based conformational selection allosteric network of the free PDZ2 domain (**b**), eNOE-based conformational selection allosteric network of the RA-GEF2 complexed PDZ2 domain (**c**), allosteric network based on evolutionary coupling in the family of the PDZ domains^48^ (**d**), allosteric network based on MD simulation^46^ (**e**), and allosteric network based on methyl relaxation data^7^ (**f**).

We define the allosteric pathway or network within a protein as an uninterrupted structural area composed of a set of atoms that are non-locally interconnected with each other^48^. The interconnection is not further defined and can be of dynamical, energetic, or structural nature. In the present study, the structural nature of the allosteric network has been investigated in the PDZ2 domain. In a comparison between the various proposed and published allosteric pathways of the PDZ2 domain under study here, the methods used need to be investigated briefly. (i) The multi-state structure determination presented shows a direct measure of a structural correlation network between states that interchange on the time scale of larger than 10 ns to micro or low milliseconds. (ii) Dynamics analysis using NMR-based relaxation measurements yields a local exchange rate between states. Similar rates for parts of the protein are then indicative of a correlated dynamical network between distinct structures. Dynamics can, in principle, be measured from ps to milliseconds. (iii) Molecular dynamics (MD) simulations are usually of a short time scale (i.e., in the ns range as exemplified with the PDZ2 domain having 16 replicas of a 2 ns MD trajectory)^47^. (iv) The evolutionary approach differs from the others as it does not only evaluate a single hPTP1E PDZ2 domain but a whole family of the PDZ domains. This makes this approach sensitive to a conserved allosteric interaction among all PDZ domains, which could, for example, be ligand binding. Indeed, when the various methods are applied to PDZ2 shown in Fig. 6, the allosteric pathway deduced from evolutionary data of the PDZ domain^48^ resembles the eNOE-based induced-fit allosteric network with the ligand-binding site and α-helix 1. In contrast, the allosteric pathways deduced from MD data^46^ and methyl relaxation data^7^ resemble rather the eNOE-based structural network of the apo PDZ2 covering the PDZ binding site with β-strands 2 and 3 and α-helix 2 (Fig. 6). Hence, in the case of the PDZ2 domain, the multi-state structure elucidation appears to cover well the structural aspect of allostery, and while this at atomic resolution, the dynamic nature must be measured kinetically or with relaxation measurements.

### On the multi-level allosteric mechanism of the PDZ2 domain

The multi-state structures of the apo and holo form of the PDZ2 domain indicate three levels of protein allostery (Supplementary Fig. 14). The structural correlations of the apo form comprising an extensive structural correlation network between an open ligand-welcoming and a closed ligand-obstructive state encompassing ~60% of the entire domain are in line with the presence of a conformational selection allostery mechanism of ligand binding (Fig. 5). In particular, the ligand-binding site with α-helix 2 and β-strand 2, and β-strand 3 are involved. Then, the ligand binding to the open state induces an extensive conformational change covering ~25% of the protein, including again the binding site by definition as well as most prominently α-helix 1 and β-strand 4. Interestingly this conformational change reduces the extensive allosteric network of the apo form by α-helix 2 in the complex. Moreover, while both allosteric networks comprise the binding site, they are structurally distinct also within the binding site. This can be exemplified with the two binding groove obstructing sidechains Lys38 and Lys72 (Fig. 5) in the open state 1 of apo, which overlap in the closed state 2 of apo with their orientation in the complex, while the complex shows itself again two distinct orientations. The structural change of α-helix 1 upon complex formation may be involved in the interdomain interface of the multidomain hPTP1E, but this idea remains speculative at this point.

Overall, the well-studied PDZ2 domain showcases the power of NMR with high accuracy afforded by eNOEs. This opens up the possibility to solve multiple protein states at atomic resolution under physiological conditions to study protein allostery. It elucidated a three-level allosteric network at atomic resolution and pinpoints the existence of rather extensive and sophisticated structural correlation networks of residues that, in principle, could be used for ligand binding regulation or signaling. In the context of the system of interest, the PDZ2 allosteric binding mechanism was found to be combined with the broadly accepted induced-fit and conformational selection mechanisms. In more general terms, the presented work validates also in part previously reported allosteric indicators using a genetic algorithm^48^, experimental data^7^, and MD simulation^46^. We expect that the presented findings of a complex structural landscape of a protein comprising correlated structural states, which interchange dynamically in the microsecond time range, are present in almost any biomolecular system awaiting to be explored.

## Methods

### Expression and purification of PDZ2 Domain

The DNA shuttle vector harboring the PDZ2 sequence from human tyrosine phosphatase 1E (hPTP1E) was used for bacterial expression of the PDZ2 domain. The gene of interest included an N-terminal polyhistidine tag separated by an HRV-3C protease cleavage site. Expression and purification was carried out according to the previously reported procedures with minor modifications^7,64^. Protein expression was performed in BL21 (DE3) *Escherichia coli* cells and induced with 1 mM IPTG after reaching an OD_600_ of 0.8. Stable isotope labeling was performed by resuspending cells in growth media supplemented with ^15^N-enriched ammonium chloride and ^13^C-enriched glucose. After overnight incubation with shaking, cells were harvested, resuspended, and lysed with a microfluidizer. The protein of interest was purified from the lysate with Ni-NTA chromatography. Then the polyhistidine tag was cleaved with HRV-3C protease and the protein of interest was further separated by passing through the Ni-NTA column. The eluted protein was concentrated at 2 mM and the buffer was exchanged for the desired buffer for NMR (150 mM sodium chloride and 50 mM phosphate buffer at pH 6.8). A peptide comprising the eight C-terminal residues from Rap Guanine Nucleotide Exchange Factor (RA-GEF2) (Ac-ENEQVSAV–COOH, BACHEM) was added to the final sample at a concentration of 2 mM (1:1) for measurements of the PDZ2 domain bound to the ligand.

### NMR experiments

The NMR measurements were performed on 600, 700, and 900 MHz Bruker spectrometers equipped with a triple resonance cryoprobe at 298 K. Processing and analysis of all NMR spectra were done with NMRPipe^65^, Bruker Topspin 4.0, and XEASY^66^. The PDZ2-peptide ligand titration experiments were performed by acquiring [^1^H,^15^N]-HSQC spectra at 298 K, for a uniformly ^15^N-enriched PDZ2 domain both for a free form and bound to the peptide supplied in a twofold excess. The rotational correlation times τ_c_ was calculated to be 6.9 ns for the apo PDZ2 and 6.4 ns for the PDZ2 domain in complex with RA-GEF2 peptide from the ^15^N-relaxation experiments as described previously^67^. It was also optimized using a systematic screening approach with the goal of target function minimization in the structure calculations. NOE-buildups were recorded with [^15^N,^13^C]–resolved [^1^H,^1^H]–NOESY with 40 (t_1_,max(^15^N) = 14:4 ms = t_1_,max(^13^C) = 7:6 ms)* 200 (t_2_,max(^1^H) = 22:0 ms)*1024 (t_3_,max(^1^H) = 102:5 ms) complex points, an interscan delay of 0.6 s, four scans per increment and mixing times of 8, 16, 24, 32, 40, 48, and 80 ms. The data were zero-filled to 1024 points in the direct proton dimension, to 128 points in the ^15^N/^13^C–dimension and to 512 points in the indirect proton dimension, processing was done with a squared cosine window function for the proton dimensions and a cosine window function for the ^15^N/^13^C–dimension. Scalar couplings ^1^*J*_HNHα_ were recorded as previously described in ref. 68 from a series of intensity-modulated HMQC spectra with 80(t_1,max_(^15^N) = 28.2 ms)*512(t_2,max_(^1^H) = 52.3 ms) complex points with an interscan delay of 1 s and 32 scans per increment. Scalar couplings ^3^*J*_HαHβ_ were recorded as previously described^69^ from a 3D ^13^Cα-separated HACAHB-COSY experiment with 50(t_1,max_(^13^C) = 14.2 ms)* 54 (t_2,max_(^1^H) = 7.5 ms)* 2048 (t_3,max_(^1^H) = 204.9 ms) complex points, 16 scans per increment and 1 s of interscan delay. Scalar couplings for aromatic sidechain heavy atoms ^1^*J*_NCγ_ and ^1^*J*_COCγ_ were recorded as previously described^70^ from intensity-modulated HSQC spectra with 200(t_1,max_(^15^N) = 150.0 ms)*512(t_2,max_(^1^H) = 51.2 ms) complex points, and interscan delay of 1.2 s and 16 scans per increment for ^1^*J*_NCγ_ couplings and with 100(t_1,max_(^15^N) = 75.0 ms)*512(t_2,max_(^1^H) = 51.2 ms) complex points, and interscan delay of 1 s and 32 scans per increment for ^1^*J*_COCγ_ couplings.

### ^15^N-relaxation measurements and dynamics analysis

^15^N *R*_1*ρ*_, *R*_1_, and ^15^N{^1^H}-NOE relaxation data was measured at ^1^H fields of 600 and 900 MHz for both apo and complex PDZ2 domain. For the ^15^N *R*_1_ relaxation measurements, a series of interleaved experiments^17^ with relaxation delays of 0, 160, 320, 480, 640, 800, 960, 1120, 1280, and 1440 ms, were collected. For the ^15^N *R*_1*ρ*_ relaxation measurements, the relaxation delays were 15, 20, 30, 40, 50, 60, 70, and 80 ms, under a spin-lock field of 2200 Hz. The number of complex points recorded in the direct proton dimension and the indirect ^15^N dimension were 1024 and 320, respectively. The interscan delay was chosen to be 2 s. The relaxation decay curves were fit using NMRPipe3 and the obtained relaxation rates, as shown in Supplementary Tables 4, 5, were analyzed using the Model-free analysis routine^18^ present in the software package RELAX^19–21^. The optimal model selected by the model-free analysis was used to determine the value of the chemical exchange rate, *R*_ex_, given in Supplementary Fig. 12 for the field frequency of 900 MHz.

### Structure calculation

Single- and multi-state protein structure calculations were performed according to the previously reported procedure^29,51,52^ using eNORA2^24,25^ within CYANA^19^. Distance extraction with eNORA2 used for spin diffusion correction an initially derived standard single-state NMR structure for apo, and in the case of the holo PDZ2, the crystal structure with PDB code 3LNY [10.2210/pdb3LNY/pdb]^25,44^. Lower and upper distance restraints from eNOEs, backbone, Hβ, and aromatic sidechain scalar couplings were used as inputs for the structure calculation. Calculations were done with 150’000 torsion angle dynamics steps for 1000 conformers by simulated annealing. Identical heavy atoms from multi-state conformers were kept together by a potential well with a bottom width of 1.2 Å, as previously described in ref. 71. No further refinement was done explaining at least in part the non-optimal Ramachandran statistics.

### eNOE dataset for PDZ2 domain in the apo form

An exhaustive set of experimental restraints for the PDZ2 in the apo form consisted of 1143 unidirectional distance restraints, 410 bidirectional distance restraints with the highest precision of 0.1 Å and 130 scalar couplings, which results in 17 restraints per residue as summarized in Supplementary Table 1.

### eNOE dataset for PDZ2 domain in holo form

For the PDZ2 domain in complex with RA-GEF2 peptide, the experimental set of restraints consisted of 995 unidirectional distance restraints, 489 bidirectional distance restraints and 130 scalar couplings, which results in 16 restraints per residue as summarized in Supplementary Table 2.

### Reporting summary

Further information on research design is available in the Nature Research Reporting Summary linked to this article.

## Supplementary information

Supporting Information

Reporting Summary

## Data Availability

The protein structure data that support the findings of this study have been deposited in the Protein Data Bank under accession codes 3LNX [10.2210/pdb3LNX /pdb] (apo PDZ2) and 3LNY [10.2210/pdb3LNY/pdb] (PDZ2 bound to RA-GEF2 peptide). The protein structure data that was generated in this study have been deposited in the Protein Data Bank under accession codes 7QCX [10.2210/pdb7QCX/pdb] (apo PDZ2) and 7QCY [10.2210/pdb7QCY/pdb] (PDZ2 bound to RA-GEF2 peptide) and in the Biological Magnetic Resonance Bank under the accession codes 34688 [https://bmrb.io/data_library/summary/index.php?bmrbId=34688] and 34689 [https://bmrb.io/data_library/summary/index.php?bmrbId=34689].
