# Supplementary material for: Atomic resolution protein allostery from the multi-state structure of a PDZ domain
Source: Nat Commun. 2022 Oct 20;13:6232. doi: 10.1038/s41467-022-33687-x (PMC9584909; doi:10.1038/s41467-022-33687-x)
Supplement: Supplementary file 1 — Supporting Information [file 41467_2022_33687_MOESM1_ESM.pdf]

Supporting Information for the manuscript

Atomic resolution Protein Allostery from the multi-state Structure of a PDZ Domain

Dzmitry Ashkinadze, Harindranath Kadavath, Aditya Pokharna, Celestine N. Chi, Michael Friedmann, Dean Strotz, Pratibha Kumari, Martina Minges, Riccardo Cadalbert, Stefan Königl, Peter Güntert, Beat Vögeli*, Roland Riek*

SUPPLEMENTARY TABLES

**Supplementary Table 1** Structural statistics and CYANA input data for the apo PDZ2 domain.		
**NMR distance and dihedral constraints**		
**Distance constraints**		
Total eNOEs	1484	
eNOEs from one pathway	996	
eNOEs from two pathways	488	
Intra-residue, $\|i-j\|=0$	545	
Sequential, $\|i-j\|=1$	373	
Short-range, $\|i-j\|\leq 1$	918	
Medium-range, $3<\|i-j\|<5$	162	
Long-range, $\|i-j\|\geq 5$	404	
**Dihedral angle restraints**		
$^3JHN_\alpha$ scalar coupling	65	
$^3JH_\alpha H_\beta$ scalar coupling	55	
^3JHNCG scalar coupling (aromatic)	5	
$^3JHNCOCG$ scalar coupling (aromatic)	5	
$^{13}C_\alpha$ chemical shifts	86	
	One-state ensemble	Two-states ensemble
**Structure statistics**		
Average CYANA target function value (Å)	35.88 ± 0.29	7.36 ± 0.17
Violations		
Distance constraints (>0.5 Å)	12	0
Dihedral angle constraints (>5°)	0	0
**Deviations from idealized geometry**		

RMSD (Å)		
Backbone to mean	0.50 ± 0.09	0.64 ± 0.08
Heavy atoms to mean	0.90 ± 0.12	1.20 ± 0.10
**RMSD to X-ray structure (PDB ID 3LNX [10.2210/pdb3LNX/pdb]) / Å**		
Backbone	0.90	1.14
Heavy atoms	1.30	1.77

**Supplementary Table 2** Structural statistics and CYANA input data for the PDZ2 domain in complex with RA-GEF2 peptide.		
**NMR distance and dihedral constraints**		
**Distance constraints**		
Total eNOEs	1547	
eNOEs from one pathway	1137	
eNOEs from two pathways	410	
Intra-residue, $\|i-j\|=0$	526	
Sequential, $\|i-j\|=1$	410	
Short-range, $\|i-j\|\leq 1$	936	
Medium-range, $1<\|i-j\|<5$	204	
Long-range, $\|i-j\|\geq 5$	407	
**Dihedral angle restraints**		
$^{3}JHN_{\alpha}$ scalar coupling	65	
$^{3}JH_{\alpha}H_{\beta}$ scalar coupling	55	
$^{3}JHNCG$ scalar coupling (aromatic)	5	
$^{3}JHNCOCG$ scalar coupling (aromatic)	5	
$^{13}C_{\alpha}$ chemical shifts	80	
	One-state ensemble	Two-states ensemble
**Structure statistics**		
Average CYANA target function value (Å)	48.31 ± 1.47	13.58 ± 0.36
Violations		
Distance constraints (>0.5Å)	33	1
Dihedral angle constraints (>5°)	0	0

Deviations from idealized geometry		
RMSD (Å)		
Backbone to mean	0.39 ± 0.10	0.62 ± 0.06
Heavy atoms to mean	0.91 ± 0.12	1.19 ± 0.10
**RMSD to X-ray structure (PDB ID 3LNY [10.2210/pdb3LNY/pdb]) / Å**		
Backbone	1.53	1.41
Heavy atoms	2.20	1.99

**Supplementary Table 3** Ramachandran statistics of the apo and holo PDZ two-state structures.		
	Apo	Complex
Most favored regions	69.6%	68.0%
Additionally allowed regions	28.0%	28.8%
Generously allowed regions	1.8%	2.3%
Disallowed regions	0.5%	0.9%

Supplementary Table 4 R1ρ, R1, and $^{15}N\{^{1}H\}$-NOE rates of apo PDZ2 domain with the corresponding errors.

Residue No.	Relaxation Rates (600MHz) [s^{-1}]						Relaxation Rates (900MHz) [s^{-1}]					
#	R1	ΔR1	R2	ΔR2	NOE	ΔNOE	R1	ΔR1	R2	ΔR2	NOE	ΔNOE
2	1.60	0.05	9.49	0.08	0.67	0.03	1.06	0.01	12.00	0.18	0.68	0.03
4	1.61	0.06	11.23	0.12	0.70	0.02	1.07	0.02	14.57	0.26	0.80	0.04
5	1.64	0.03	10.39	0.07	0.64	0.01	1.19	0.01	12.84	0.14	0.77	0.02
6	1.55	0.02	4.06	0.02	0.03	0.01	1.51	0.01	4.58	0.03	0.40	0.01
7	1.58	0.05	12.50	0.10	0.78	0.02	1.04	0.02	16.42	0.24	0.84	0.03
8	1.42	0.03	11.97	0.08	0.75	0.02	0.93	0.01	15.36	0.16	0.84	0.03
9	1.46	0.04	11.64	0.09	0.72	0.02	0.94	0.01	14.38	0.21	0.85	0.04
10	1.22	0.03	11.69	0.09	0.81	0.03	0.90	0.01	14.85	0.22	0.81	0.03
11	1.53	0.04	11.13	0.09	0.73	0.02	0.98	0.01	14.47	0.21	0.84	0.03
12	1.45	0.04	11.00	0.08	0.69	0.02	0.98	0.01	14.57	0.18	0.74	0.03
13	1.54	0.04	10.59	0.09	0.61	0.02	1.01	0.02	13.93	0.21	0.82	0.04
14	1.47	0.04	10.70	0.09	0.72	0.02	1.05	0.01	13.20	0.19	0.82	0.03
16	1.62	0.09	21.55	0.26	-	-	-	-	-	-	-	-
17	1.39	0.03	11.51	0.08	0.71	0.02	1.05	0.01	15.07	0.18	0.81	0.03
20	1.34	0.04	13.04	0.12	0.68	0.02	1.10	0.03	17.57	0.52	0.96	0.06
21	1.47	0.04	16.08	0.13	0.76	0.02	0.95	0.02	24.33	0.43	0.77	0.05
22	1.52	0.09	13.20	0.53	0.78	0.04	1.08	0.04	15.55	0.62	1.05	0.10
23	1.52	0.05	12.11	0.10	0.77	0.02	0.99	0.02	15.63	0.26	0.83	0.04
24	1.52	0.08	10.61	0.13	0.82	0.03	1.05	0.03	13.47	0.35	0.83	0.06
25	1.39	0.17	14.99	0.56	0.86	0.08	-	-	-	-	-	-
26	1.57	0.05	11.32	0.12	0.74	0.03	1.13	0.03	14.64	0.34	0.82	0.05
27	1.75	0.10	18.40	0.23	0.64	0.04	1.20	0.04	30.34	1.09	0.76	0.08
28	1.47	0.10	14.82	0.28	0.79	0.05	1.05	0.05	24.41	1.17	0.72	0.08
30	1.42	0.05	15.53	0.14	0.71	0.03	1.08	0.03	24.12	0.54	0.91	0.07
31	1.71	0.10	14.89	0.28	0.62	0.04	1.16	0.05	24.02	1.06	0.67	0.08
32	-	-	-	-	-	-	-	-	-	-	0.78	0.04
33	1.60	0.05	10.31	0.13	0.66	0.02	1.19	0.02	12.45	0.28	0.79	0.04
35	1.60	0.02	13.56	0.15	0.06	0.01	1.57	0.01	3.87	0.03	0.45	0.01
36	1.53	0.06	12.28	0.13	0.76	0.03	0.93	0.02	15.54	0.32	0.81	0.04
37	1.52	0.05	11.61	0.10	0.80	0.02	0.95	0.02	14.39	0.23	0.79	0.03
38	1.60	0.08	10.91	0.16	0.72	0.03	1.12	0.03	15.01	0.45	0.88	0.05
39	1.34	0.03	10.11	0.06	0.78	0.02	1.02	0.01	12.45	0.12	0.85	0.03
40	1.56	0.02	10.00	0.07	0.77	0.01	1.02	0.01	10.99	0.18	0.85	0.03
41	1.47	0.05	10.62	0.11	0.81	0.03	1.06	0.02	13.33	0.26	0.82	0.04
43	1.48	0.05	11.29	0.09	0.74	0.02	0.92	0.02	14.10	0.20	0.81	0.03
44	1.39	0.03	10.58	0.09	0.77	0.02	1.08	0.01	13.92	0.20	0.79	0.03
45	1.51	0.04	13.36	0.09	0.80	0.02	0.99	0.02	17.19	0.31	0.82	0.04
46	1.34	0.03	12.44	0.12	0.77	0.02	1.02	0.03	16.32	0.57	0.84	0.06
47	1.54	0.05	13.12	0.10	0.81	0.02	0.99	0.02	17.48	0.30	0.89	0.04
48	1.31	0.03	12.29	0.08	0.78	0.02	0.94	0.01	15.90	0.20	0.89	0.03
49	1.29	0.03	12.56	0.07	0.72	0.02	0.99	0.01	16.15	0.18	0.86	0.03

50	-	-	-	-	-	-	0.94	0.02	15.95	0.27	0.81	0.03
51	1.39	0.03	10.37	0.09	0.78	0.02	1.01	0.01	13.18	0.17	0.86	0.03
53	1.59	0.03	11.21	0.08	0.76	0.01	1.01	0.01	14.56	0.17	0.86	0.03
55	1.47	0.05	11.96	0.11	0.73	0.02	0.96	0.02	15.04	0.24	0.86	0.04
56	1.48	0.02	10.99	0.07	-	-	-	-	-	-	-	-
58	1.60	0.05	11.67	0.11	0.82	0.02	0.98	0.02	15.02	0.28	0.84	0.04
59	1.59	0.07	11.97	0.14	0.77	0.03	0.99	0.02	15.23	0.33	0.86	0.05
61	1.54	0.01	10.94	0.06	-	-	-	-	-	-	-	-
62	1.59	0.07	11.93	0.13	0.73	0.03	1.02	0.02	14.67	0.39	0.83	0.04
63	-	-	-	-	0.77	0.03	1.09	0.02	15.48	0.34	0.81	0.04
64	1.40	0.03	10.72	0.08	0.83	0.03	1.07	0.01	13.28	0.17	0.80	0.03
66	1.39	0.03	10.94	0.11	0.51	0.02	1.01	0.02	12.02	0.20	0.77	0.04
67	1.53	0.03	11.12	0.06	0.66	0.02	1.10	0.01	14.77	0.16	0.76	0.03
68	1.50	0.08	12.02	0.18	-	-	-	-	-	-	-	-
69	1.25	0.03	12.90	0.08	0.82	0.03	1.01	0.02	16.56	0.26	0.80	0.03
70	1.52	0.06	11.35	0.11	0.79	0.03	1.01	0.02	14.48	0.24	0.92	0.05
71	1.76	0.07	11.34	0.10	0.76	0.03	1.13	0.02	14.51	0.25	0.80	0.03
72	1.62	0.06	11.28	0.09	0.75	0.03	1.06	0.02	14.06	0.29	0.91	0.05
73	1.47	0.04	10.88	0.07	0.78	0.02	1.06	0.01	13.54	0.15	0.81	0.03
74	1.66	0.05	11.34	0.07	0.77	0.02	1.11	0.01	14.48	0.19	0.81	0.03
75	1.48	0.07	11.09	0.06	0.73	0.01	1.03	0.01	12.66	0.20	0.81	0.03
76	1.50	0.03	11.94	0.08	0.62	0.02	1.45	0.02	5.43	0.09	0.84	0.04
77	1.48	0.02	11.04	0.07	0.74	0.01	1.06	0.01	13.72	0.13	0.84	0.03
78	1.44	0.03	10.67	0.09	0.67	0.02	1.07	0.01	12.67	0.18	0.80	0.03
79	1.47	0.02	4.70	0.04	0.09	0.01	1.34	0.01	6.35	0.05	0.39	0.02
80	1.24	0.01	10.37	0.07	0.40	0.01	1.00	0.01	12.21	0.10	0.76	0.03
81	1.13	0.03	7.80	0.08	0.59	0.02	0.91	0.01	9.84	0.12	0.58	0.03
82	-	-	-	-	-	-	1.06	0.02	11.10	0.21	-	-
84	1.23	0.03	10.81	0.06	0.80	0.02	0.99	0.01	13.31	0.13	0.87	0.03
85	1.57	0.05	10.94	0.10	0.79	0.02	0.99	0.02	14.33	0.24	0.82	0.03
86	1.54	0.05	12.09	0.10	0.71	0.02	0.98	0.02	15.74	0.26	0.82	0.04
87	1.51	0.04	11.95	0.10	0.79	0.02	0.96	0.02	15.21	0.23	0.90	0.03
88	1.33	0.04	12.88	0.12	0.81	0.03	0.93	0.02	15.51	0.24	0.89	0.04
89	1.56	0.05	12.27	0.12	0.74	0.03	0.99	0.02	15.90	0.28	0.86	0.04
90	1.51	0.05	11.43	0.11	0.76	0.03	0.95	0.02	13.96	0.24	0.83	0.04
91	1.61	0.07	11.76	0.12	0.74	0.02	1.06	0.02	15.16	0.35	0.78	0.04
92	1.67	0.06	11.65	0.12	0.73	0.02	1.09	0.02	14.71	0.29	0.80	0.04
93	1.43	0.02	10.28	0.06	0.62	0.01	1.03	0.01	12.89	0.10	0.64	0.02
94	1.54	0.05	5.47	0.06	0.17	0.02	1.37	0.02	5.72	0.06	0.52	0.02
96	0.94	0.01	2.78	0.02	-0.32	0.01	1.20	0.01	2.98	0.02	0.14	0.01

**Supplementary Table 5** R1ρ, R1, and $^{15}N\{^{1}H\}$-NOE data of complex PDZ2 domain with the corresponding errors.												
Residue No.	Relaxation Rates (600MHz) [s^{-1}]						Relaxation Rates (900MHz) [s^{-1}]					
#	R1	ΔR1	R2	ΔR2	NOE	ΔNOE	R1	ΔR1	R2	ΔR2	NOE	ΔNOE
4	1.54	0.05	11.42	0.16	0.73	0.02	1.01	0.04	14.83	0.54	0.86	0.05
5	1.48	0.02	3.94	0.03	0.03	0.01	1.45	0.02	5.47	0.07	0.38	0.02
6	1.58	0.03	10.50	0.10	0.59	0.01	1.24	0.02	10.25	0.18	0.69	0.03
7	1.47	0.04	12.66	0.13	0.80	0.02	0.97	0.03	18.41	0.57	0.80	0.04
8	1.31	0.03	12.41	0.11	0.70	0.02	0.85	0.02	16.50	0.40	0.80	0.04
11	1.45	0.04	11.30	0.17	0.75	0.02	0.92	0.04	15.60	0.62	0.84	0.06
12	1.38	0.03	10.97	0.11	0.69	0.02	0.91	0.03	15.17	0.43	0.81	0.05
13	1.38	0.03	12.09	0.12	0.74	0.02	0.91	0.03	14.72	0.39	0.84	0.05
14	1.41	0.03	10.32	0.11	0.72	0.02	1.00	0.03	14.09	0.34	0.80	0.04
17	1.31	0.03	11.11	0.24	0.67	0.03	1.02	0.05	15.62	0.70	0.93	0.14
20	1.20	0.09	11.23	0.16	0.79	0.04	0.90	0.13	17.58	2.28	0.75	0.09
22	1.41	0.18	12.00	0.26	0.65	0.05	1.32	0.39	15.45	3.75	1.03	0.22
23	1.44	0.07	12.57	0.24	0.80	0.03	0.94	0.07	16.11	1.24	0.85	0.12
26	1.48	0.15	5.54	0.28	0.16	0.04	1.84	0.42	10.87	3.63	0.46	0.18
30	1.36	0.04	15.18	0.15	0.67	0.02	1.06	0.04	26.17	0.98	0.70	0.05
31	1.67	0.12	14.43	0.76	0.87	0.07	1.18	0.14	26.95	3.37	0.46	0.20
32	1.53	0.09	10.60	0.36	0.70	0.04	1.09	0.10	13.95	1.09	0.67	0.12
33	1.64	0.07	10.34	0.21	0.68	0.03	1.17	0.06	14.78	0.75	0.72	0.08
34	-	-	-	-	0.78	0.09	1.37	0.38	28.16	9.28	0.28	0.25
35	1.45	0.06	11.54	0.33	0.80	0.03	1.07	0.07	14.06	1.10	0.92	0.18
36	1.59	0.19	11.02	0.26	0.78	0.04	1.17	0.34	15.23	3.97	0.86	0.14
37	1.51	0.13	12.37	0.48	0.81	0.05	0.91	0.13	16.42	2.14	0.54	0.11
39	1.28	0.06	10.18	0.28	0.72	0.04	1.05	0.08	13.06	1.04	0.62	0.10
40	1.52	0.02	10.22	0.09	0.76	0.01	0.78	0.10	14.87	1.66	0.76	0.03
41	1.37	0.05	10.59	0.17	0.76	0.03	0.99	0.04	14.86	0.62	0.85	0.06
43	1.38	0.04	11.54	0.12	0.76	0.02	0.87	0.03	14.61	0.44	0.81	0.04
44	1.31	0.05	10.28	0.27	0.76	0.03	1.01	0.08	15.08	1.10	0.67	0.08
45	1.69	0.07	11.42	0.19	0.82	0.03	1.10	0.05	16.16	0.74	0.81	0.08
46	1.41	0.05	3.31	0.12	-0.18	0.04	1.58	0.08	4.95	0.26	0.41	0.08
48	1.24	0.02	12.61	0.09	0.79	0.02	0.88	0.02	17.12	0.45	0.89	0.04
49	1.22	0.03	12.86	0.13	0.77	0.02	0.90	0.04	19.18	0.75	0.81	0.06
50	1.19	0.03	10.65	0.14	-	-	0.88	0.04	16.69	0.67	-	-
51	1.34	0.03	10.48	0.13	0.79	0.02	0.95	0.03	14.03	0.39	0.81	0.04
52	1.52	0.01	3.45	0.03	0.15	0.01	1.53	0.02	4.55	0.06	0.44	0.02
53	1.56	0.04	10.58	0.13	0.75	0.02	1.02	0.03	15.08	0.39	0.85	0.04
56	1.45	0.02	10.86	0.09	-	-	-	-	-	-	0.81	0.03
58	1.49	0.04	11.84	0.15	0.73	0.02	0.93	0.03	16.05	0.52	0.82	0.05
59	1.55	0.09	12.20	0.28	0.78	0.04	0.93	0.07	17.07	1.17	0.75	0.09
60	1.21	0.02	12.93	0.12	0.55	0.01	0.96	0.02	13.06	0.31	0.80	0.04

61	1.59	0.02	3.58	0.05	0.03	0.01	1.54	0.03	4.70	0.08	0.43	0.02
62	1.51	0.05	12.02	0.16	0.76	0.02	1.02	0.05	16.79	0.75	0.82	0.06
63	1.59	0.05	10.41	0.15	-	0.02	1.05	0.05	16.71	0.75	0.77	0.05
64	1.35	0.03	10.86	0.11	0.73	-	0.99	0.03	14.42	0.39	0.79	0.04
66	1.43	0.02	4.40	0.05	0.02	0.01	1.38	0.02	6.00	0.09	0.43	0.02
67	1.44	0.03	11.50	0.14	0.64	0.02	1.04	0.04	14.19	0.49	0.57	0.06
69	1.18	0.04	12.80	0.18	0.89	0.04	0.94	0.07	18.96	1.12	0.87	0.09
70	1.53	0.11	12.22	0.64	0.61	0.06	1.01	0.12	16.66	2.22	-	-
71	1.81	0.30	12.05	0.39	0.87	0.08	1.97	1.02	-	-	1.07	0.30
72	1.43	0.14	12.14	0.53	0.70	0.07	1.18	0.20	19.03	3.59	1.18	0.32
73	1.39	0.05	11.04	0.17	0.80	0.03	1.04	0.05	14.16	0.67	0.74	0.08
74	1.54	0.09	11.24	0.63	0.59	0.06	1.10	0.12	13.22	1.50	1.52	0.52
75	1.49	0.11	11.03	0.69	0.71	0.04	1.00	0.05	14.84	1.29	0.57	0.15
77	0.61	0.11	-	-	-	-	0.68	0.15	-	-	0.47	0.09
78	1.45	0.05	3.70	0.14	-0.05	0.04	1.53	0.07	4.18	0.27	0.54	0.23
79	-	-	-	-	0.61	0.05	1.42	0.35	-	-	-	-
80	1.39	0.01	3.97	0.04	-0.05	0.01	1.36	0.02	5.69	0.07	0.37	0.02
81	1.07	0.02	7.56	0.10	0.52	0.02	0.86	0.03	10.54	0.26	0.61	0.04
84	1.17	0.02	10.75	0.07	0.75	0.01	0.93	0.02	14.19	0.23	0.84	0.03
85	1.49	0.04	11.12	0.14	0.76	0.02	0.95	0.03	14.88	0.49	0.87	0.05
86	1.45	0.04	12.23	0.16	0.79	0.02	0.92	0.04	17.34	0.67	0.99	0.07
87	1.44	0.04	11.83	0.13	0.75	0.02	0.93	0.03	16.65	0.45	0.83	0.04
88	1.23	0.03	12.92	0.14	0.72	0.02	0.86	0.03	17.48	0.51	0.80	0.04
89	1.47	0.05	12.58	0.17	0.80	0.03	0.91	0.04	17.59	0.76	0.88	0.06
90	1.41	0.05	11.56	0.16	0.72	0.02	0.88	0.04	15.33	0.59	0.75	0.05
92	1.61	0.07	11.40	0.20	0.81	0.03	1.06	0.06	16.32	0.89	0.74	0.06
93	1.34	0.02	10.38	0.08	0.57	0.01	1.00	0.02	14.29	0.24	0.72	0.03
94	1.49	0.04	5.28	0.09	0.19	0.01	1.37	0.03	6.46	0.14	0.48	0.03
96	0.89	0.01	2.91	0.03	-0.41	0.01	1.16	0.01	3.91	0.05	0.12	0.02

SUPPLEMENTARY FIGURES

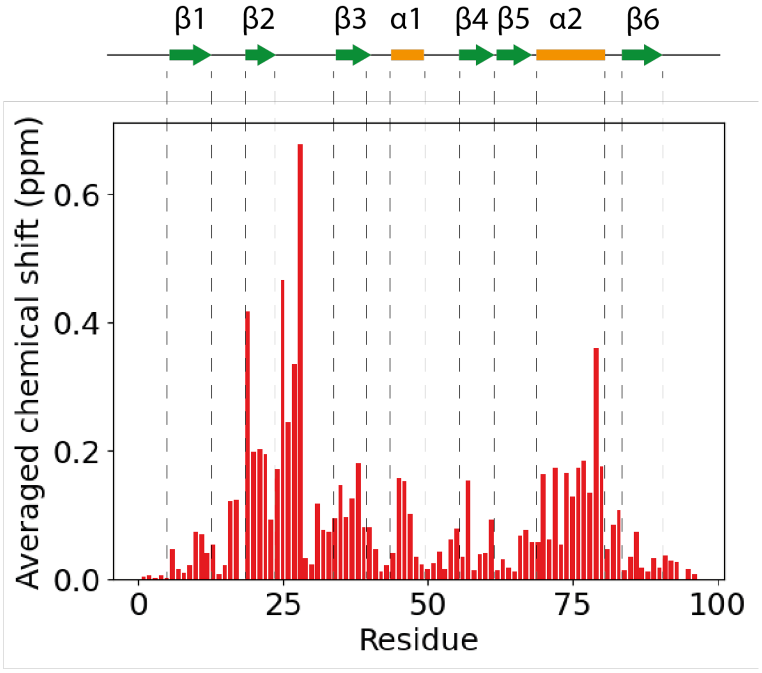

Supplementary Figure 1: Ligand-binding induced chemical shift change *versus* the amino acid sequence of PDZ2 indicates the ligand-binding site and allosteric sites. Ligand-induced ^{15}N and ^{1}H chemical shift changes (geometrically weighted with nitrogen shifts taken with weight of 0.3) measured in [^{15}N,^{1}H]-HSQC spectra of free PDZ2 domain and in a 1:1 complex with the ligand peptide RA-FEF2.

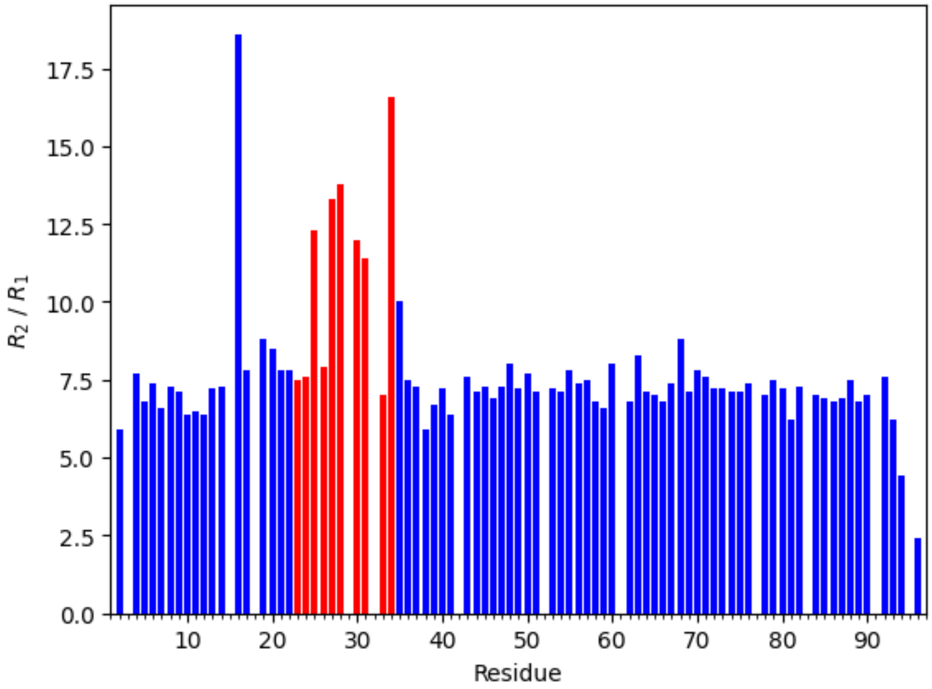

Supplementary Figure 2: The ratio between R_2 and R_1 NMR relaxation rates for the PDZ residues shows increased flexibility on the slow timescale of the PDZ loop located between the two double glycine elements Gly23-Gly24 and Gly33-Gly34 shown as red bars. Due to the increased flexibility of the PDZ loop the symmetry restraints were relaxed in the loop region.

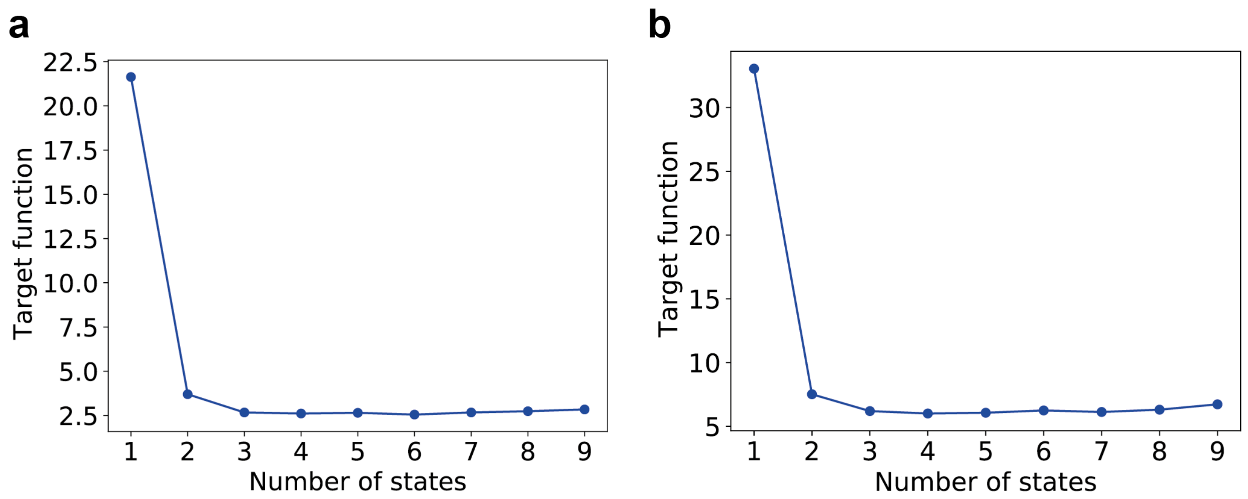

Supplementary Figure 3: Two-state validation of apo PDZ2 domain (a) and PDZ2 domain in complex with RA-GEF2 peptide (b). The CYANA target function (TF) values, which are the (weighted) sums of the squared violations of the conformational restraints, versus number of simultaneously calculated states are shown for 1-9 state structure calculations in blue. The importance of the ensemble-based structure determination is evident from the decrease of the TF with an increasing number of states and indicates that two states are sufficient to describe the experimental data well.

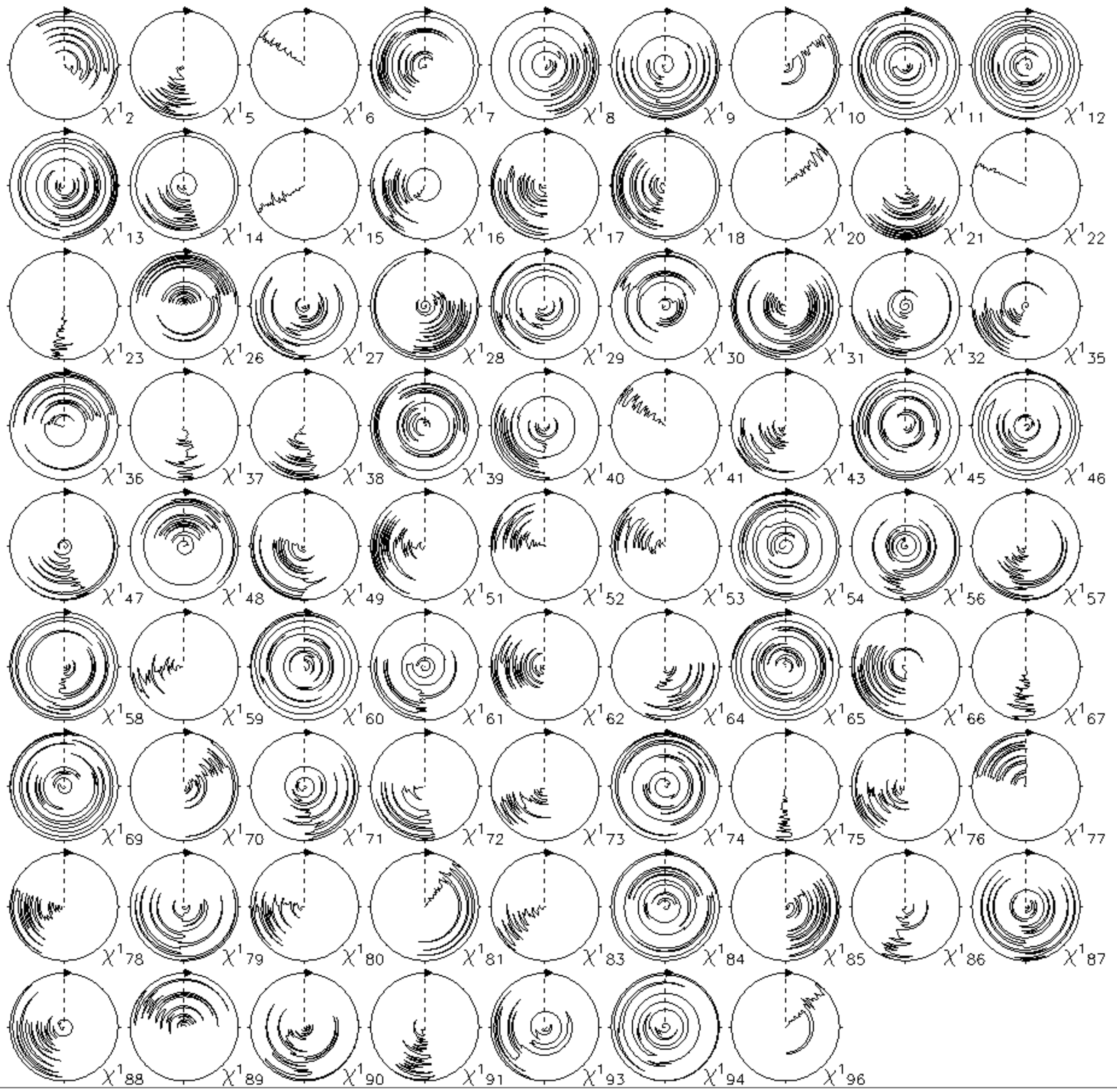

Supplementary Figure 4: Chi1 angles of all the residues of the 40 conformers of the apo PDZ2 ensemble are shown in a circular plot.

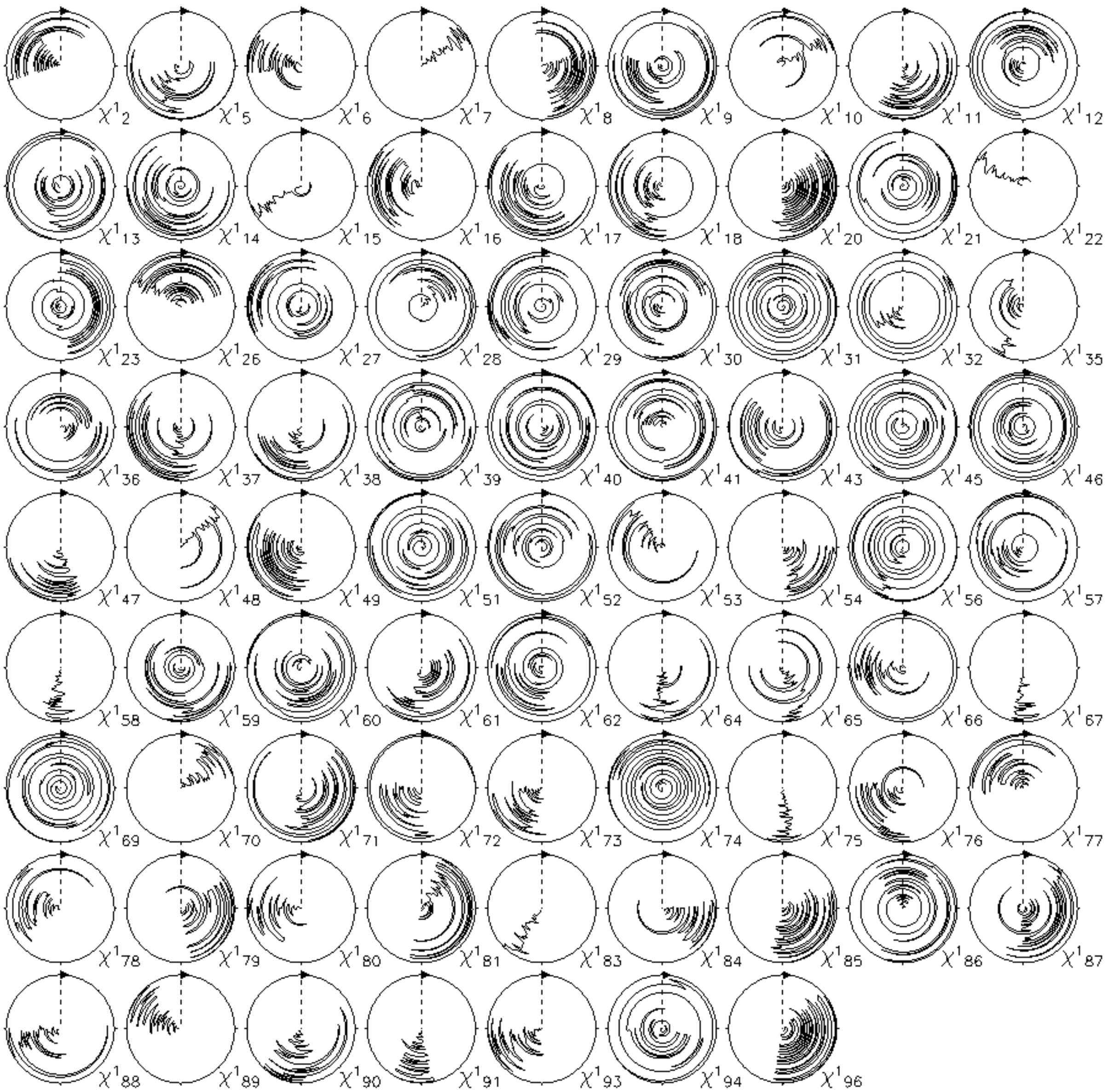

Supplementary Figure 5: Chi1 angles of all the residues of the 40 conformers of the complex PDZ2 ensemble are shown in a circular plot.

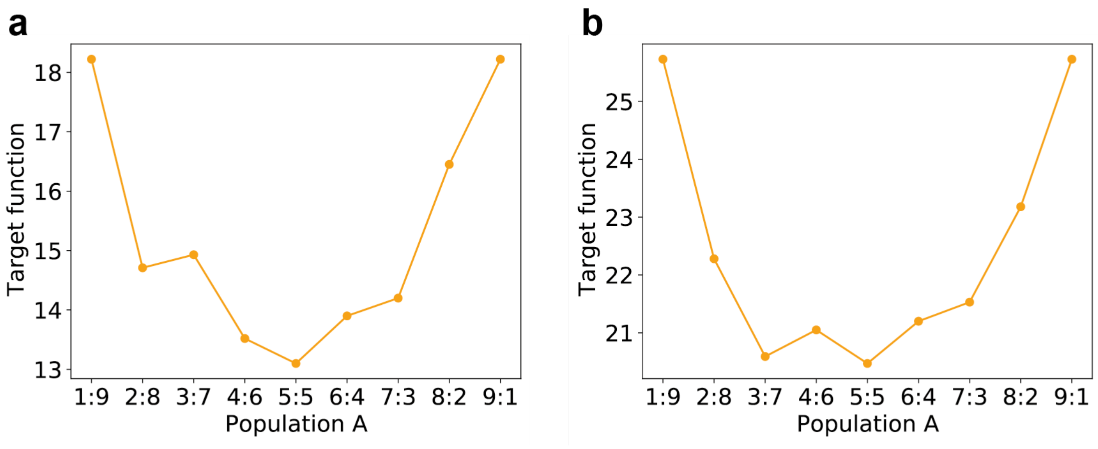

Supplementary Figure 6: Population analysis of apo PDZ2 domain (a) and PDZ2 domain in complex with RA-GEF2 peptide (b) showing that the population could be estimated from the structure calculations to be roughly 1:1. The graphs show the CYANA target function (TF) of the two-state structure calculations versus 1:9 to 9:1 populations in increments of 10%. For this a pseudo ten-state structure calculation was set up allowing only two distinct states through the application of symmetry restraints in CYANA. For example for a 2:8 ratio, a 10 state structure is calculated with artificial restraints such that two states are almost identical and the other 8 are also almost identical to each other. Because of the many additional artificial restraints stressing the structure calculation performance minimization for each population calculation 1000 conformers and 300’000 minimization steps were used.

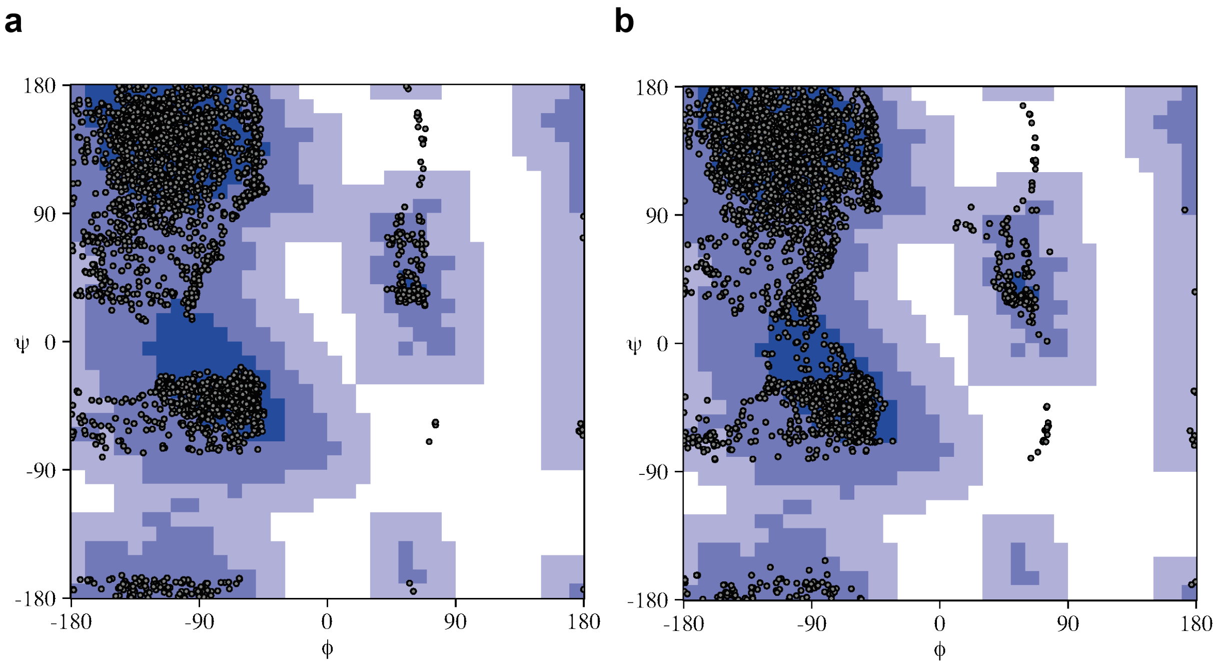

Supplementary Figure 7: Ramachandran statistics of apo PDZ2 domain (all 2*20 conformers) (a) and PDZ2 domain in complex with the RA-GEF2 peptide (all 2*20 conformers) (b).

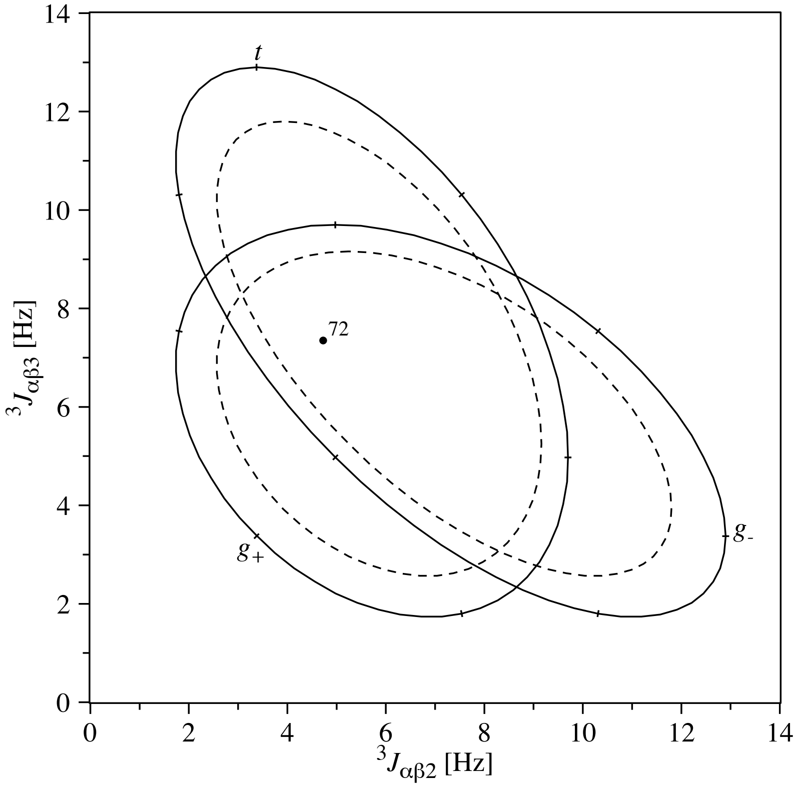

Supplementary Figure 8: Three bond scalar coupling measurements $^3J_{\alpha\beta2}$ and $^3J_{\alpha\beta3}$ of apo PDZ2 indicate that the CHI1 angle of Lys72 is in a superposition between gauche+ and trans.

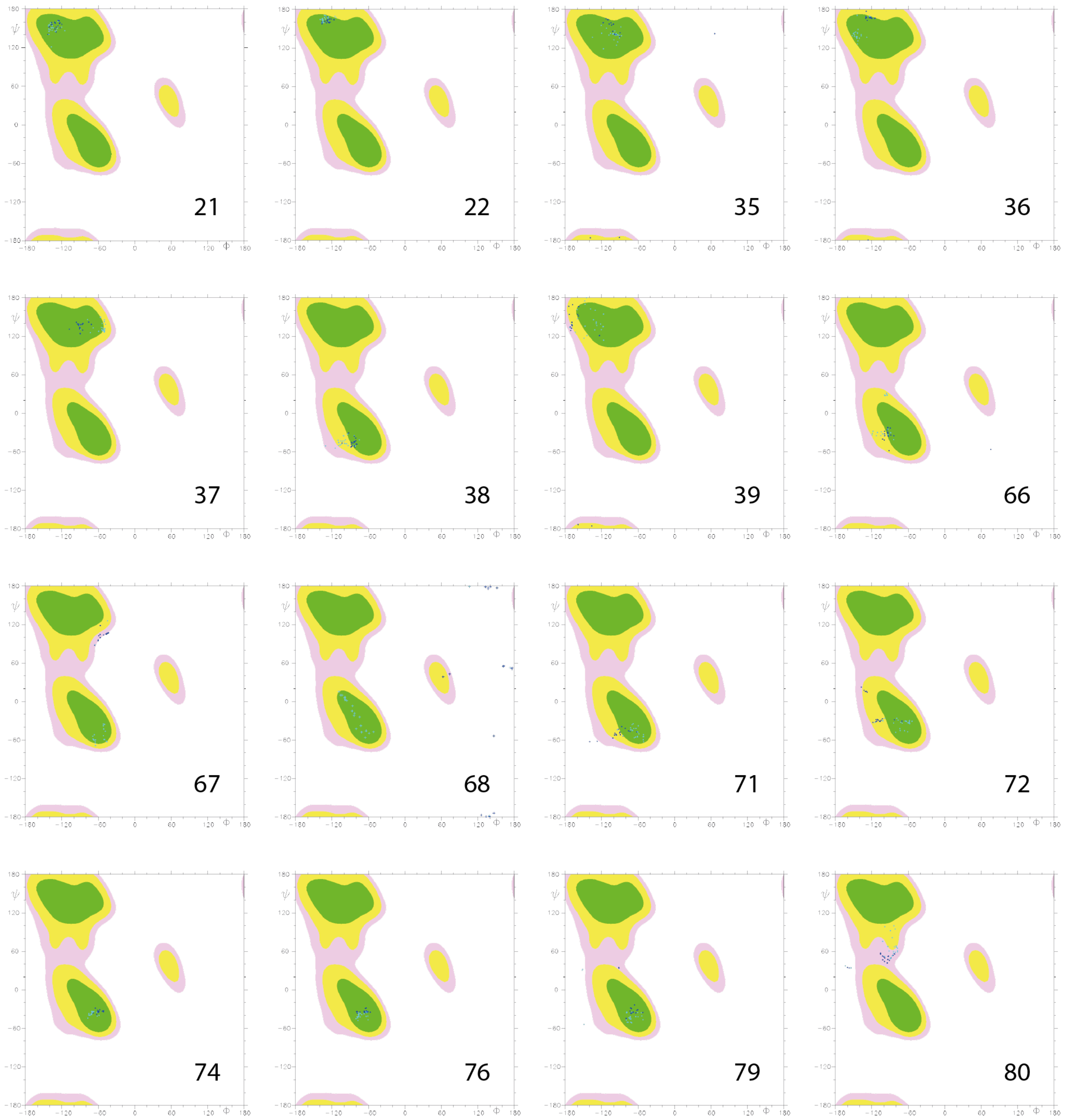

Supplementary Figure 9: Ramachandran plots of selected residues from the apo PDZ2 2*20 conformers color coded according to PDBcor selected states in cyan and blue indicate the presence of two distinct states.

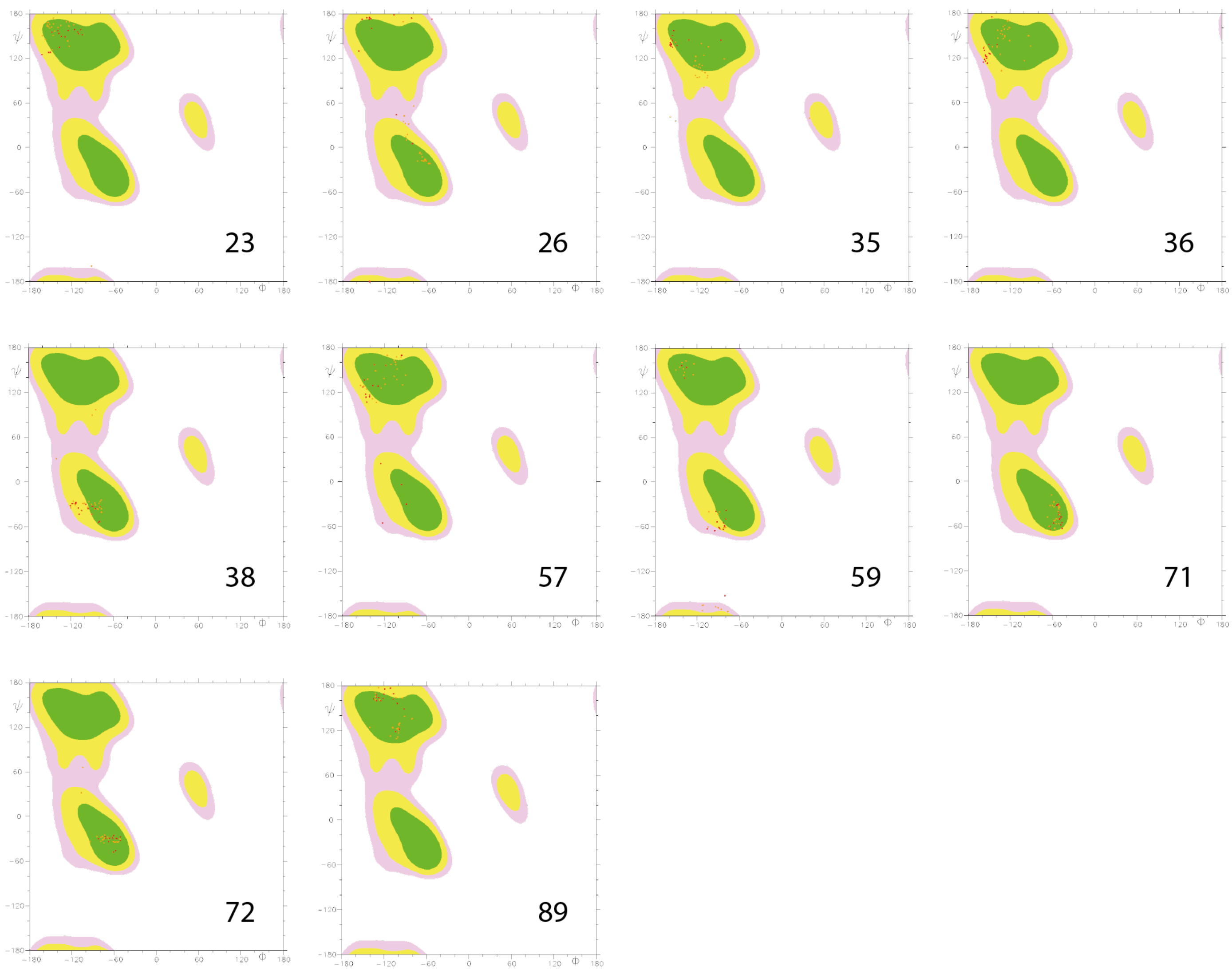

Supplementary Figure 10: Ramachandran plots of selected residues from the holo PDZ2 2*20 conformers color coded according to PDBcor selected states in cyan and blue indicate the presence of two distinct states.

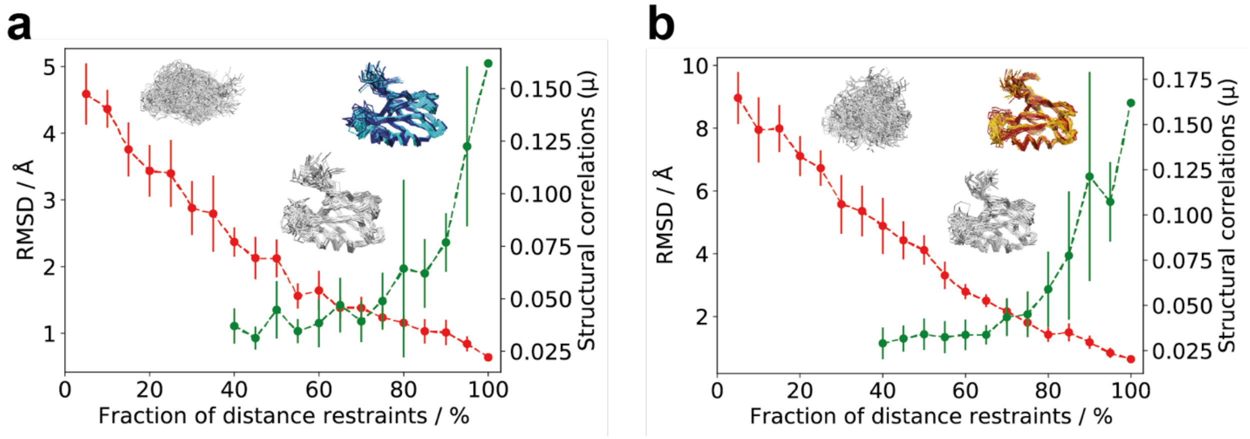

Supplementary Figure 11: A series of two-state structure calculations of the PDZ2 domain in the apo form (a) and complexed with RA-GEF2 peptide (b) was performed from subsets of distance restraints contributing to the fractions of the experimental dataset in the range from 5% up to 95% and evaluated for the ensemble RMSD (red line) and structural correlations (green line), that are visualized at their mean positions and standard deviation bars (a). In process of repetitive addition of the distance restraints both apo and holo PDZ2 domain are unfolded at 5% of distance restraints (bundle structures are illustrated top left for both), then folded above ~60% of distance restraints (middle) and individual states are well resolved above 90% of distance restraints (top right). RMSD decline showcases the acquisition of the protein fold, whereas structural correlations growth showcases the protein state separation. This analysis demonstrates that the two-state structure is overdetermined in respect to experimental restraints by ca 20%.

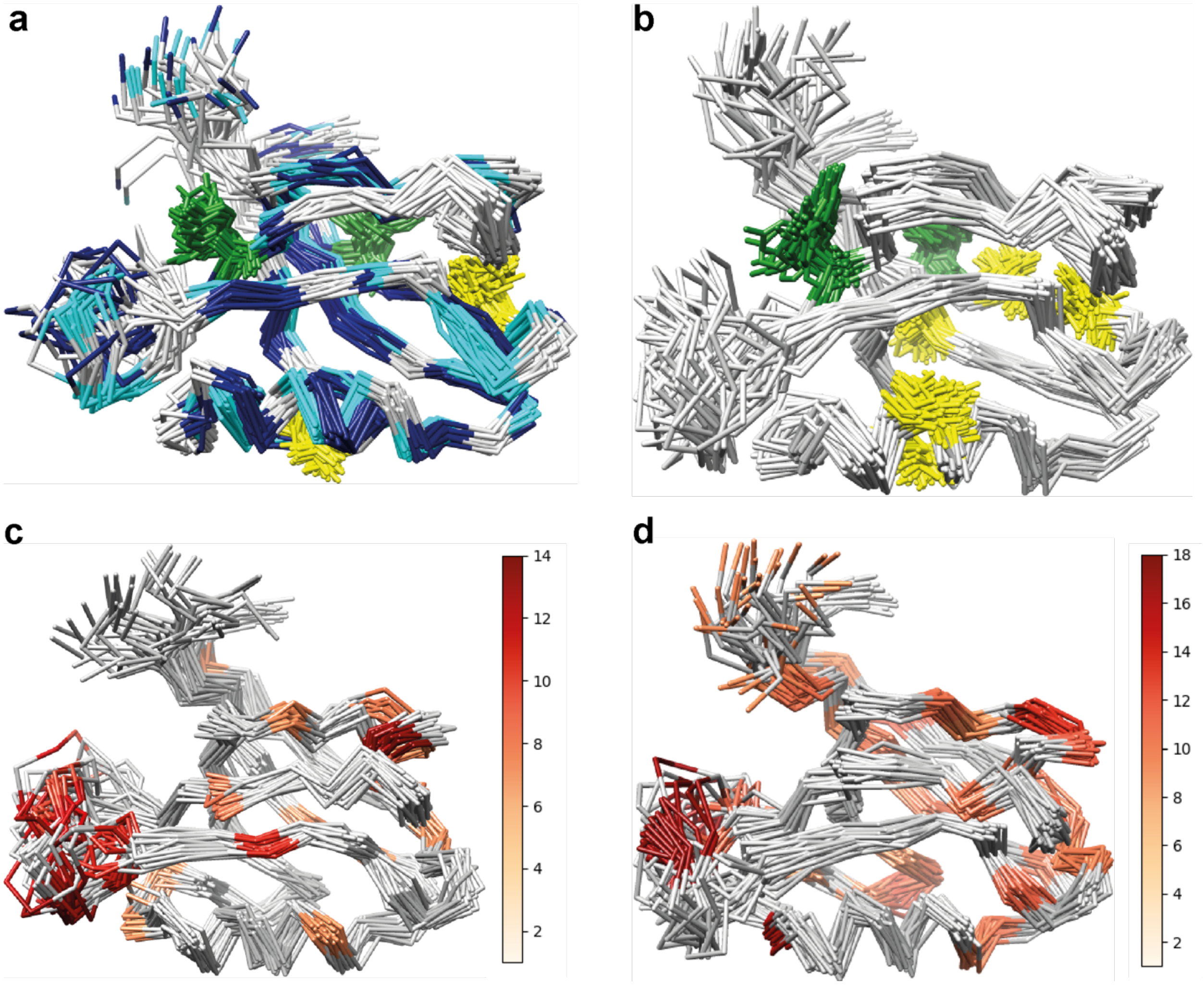

Supplementary Figure 12: Local sidechain and backbone dynamics of apo PDZ2 domain (a, c) and PDZ2 domain in complex with RA-GEF2 peptide (b, d). (a, b) Valine and Leucine residues that show for both their methyl groups the same chemical shifts are labeled by yellow sidechain. Phe7 and Tyr36 with a single aromatic protons HD1/2 and HE1/2 that requests their fast rotation are labeled green. Residues with scalar couplings $^3J_{\alpha\beta2}$ and $^3J_{\alpha\beta3}$ measured to be in a superposition between gauche+ and trans forms are colored dark blue for state 1 and cyan for state 2 (scalar couplings were measured only for the free PDZ2). (c,d) conformational exchange dynamics depicted by R_{ex} values from ^{15}N relaxation measurements at 900 MHz suggested by the software package RELAX[19-21] are indicated for apo (c) and complex (d) on top of the 3D structure. The residue color corresponds to the R_{ex} value summarized in corresponding color bar panels. Residues without the R_{ex} values are colored gray. A wide spread conformational exchange dynamics is indicated in both apo and complex. It is noted that for the complex R_{ex} may comprise contributions from exchange between ligand bound and unbound state.

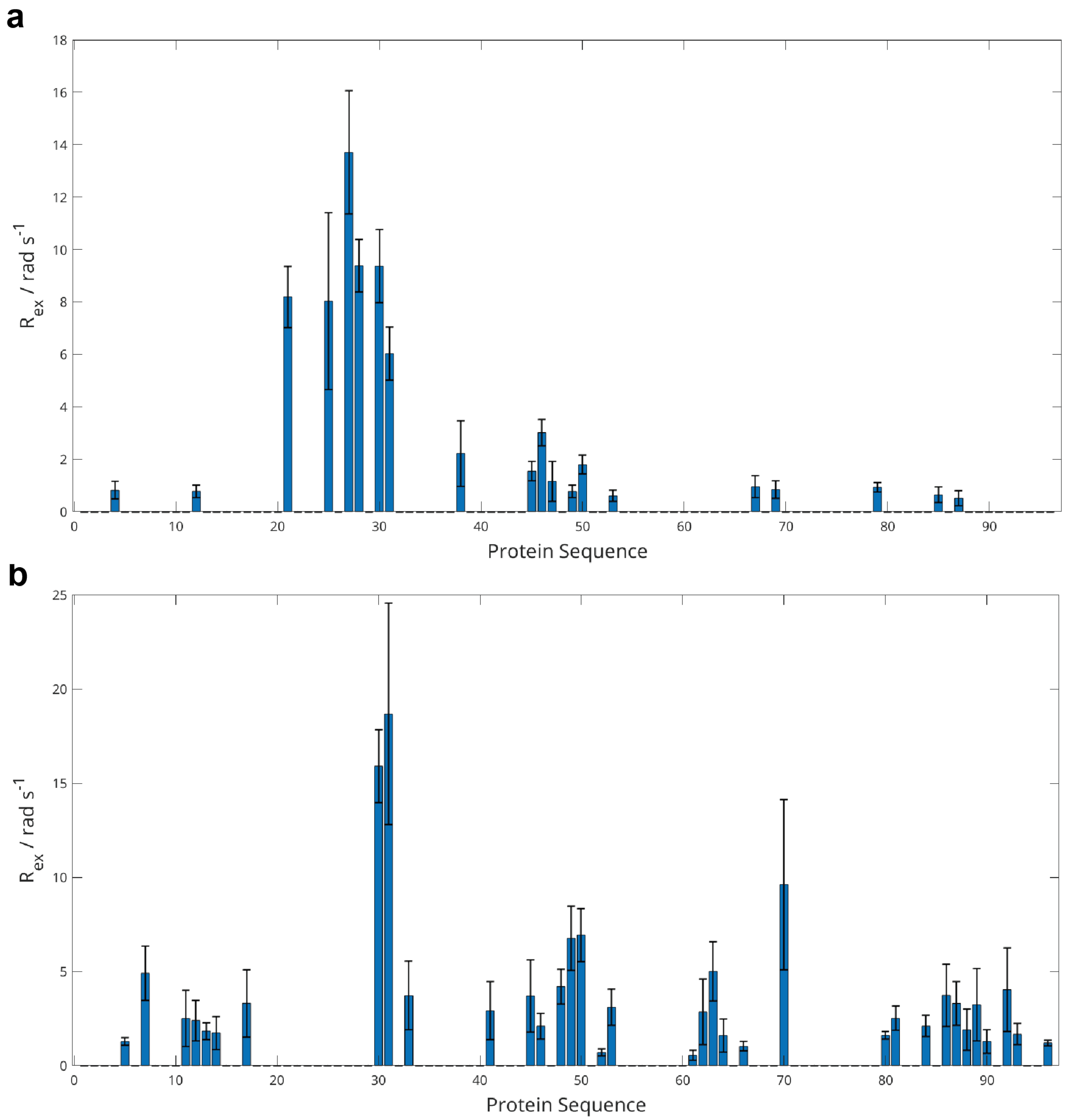

Supplementary Figure 13: Dynamic exchange contributions to the transverse ^{15}N relaxation suggested by the software package RELAX[21] for (a) apo and (b) complex PDZ2 domain are indicated in a bar plot along the amino acid sequence in units of $[rad\ s^{-1}]$. The error bars correspond to Monte Carlo simulated errors computed by the relaxGUI21. Please note that the R_{ex} values determined at 900 MHz indicate the presence of exchange, but does not give a value of the exchange rate. Furthermore, R_{ex} depends on the unknown chemical shift difference between the states of exchange and its size may vary between residues having the same exchange rate.

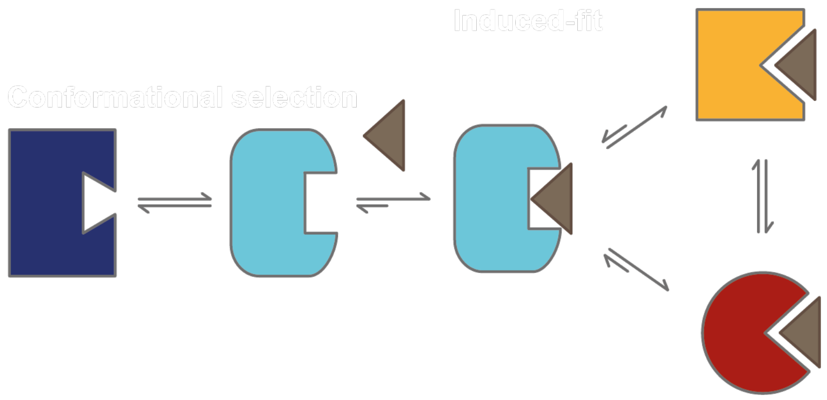

Supplementary Figure 14: Cartoon representation of a possible multi-level allosteric mechanism of the PDZ2 domain around ligand binding. The mechanism can be summarized as a conformational selection by the ligand of the open state from an equilibrium of closed and open states of apo PDZ2, followed by an induced-fit mechanism to that selected state that propagates allosteric rearrangements throughout the protein fold into the yellow and red states. The color code of the PDZ2 domain in Figure 2 is used and the ligand is indicated by a grey triangle.

SUPPLEMENTARY REFERENCES

1 Fuentes, E. J., Der, C. J. & Lee, A. L. Ligand-dependent dynamics and intramolecular signaling in a PDZ domain. *Journal of molecular biology* **335**, 1105-1115 (2004).

2 Bloem, R. *et al.* Ligand binding studied by 2D IR spectroscopy using the azidohomoalanine label. *The Journal of Physical Chemistry B* **116**, 13705-13712 (2012).

3 Delaglio, F. *et al.* NMRPipe: a multidimensional spectral processing system based on UNIX pipes. *Journal of biomolecular NMR* **6**, 277-293 (1995).

4 Bartels, C., Xia, T.-h., Billeter, M., Güntert, P. & Wüthrich, K. The program XEASY for computer-supported NMR spectral analysis of biological macromolecules. *Journal of biomolecular NMR* **6**, 1-10 (1995).

5 Kay, L. E., Torchia, D. A. & Bax, A. Backbone dynamics of proteins as studied by nitrogen-15 inverse detected heteronuclear NMR spectroscopy: application to staphylococcal nuclease. *Biochemistry* **28**, 8972-8979 (1989).

6 Kuboniwa, H., Grzesiek, S., Delaglio, F. & Bax, A. Measurement of H N-H α J couplings in calcium-free calmodulin using new 2D and 3D water-flip-back methods. *Journal of biomolecular NMR* **4**, 871-878 (1994).

7 Grzesiek, S., Kuboniwa, H., Hinck, A. P. & Bax, A. Multiple-quantum line narrowing for measurement of H. alpha.-H. beta. J couplings in isotopically enriched proteins. *Journal of the American Chemical Society* **117**, 5312-5315 (1995).

8 Hu, J.-S., Grzesiek, S. & Bax, A. Two-Dimensional NMR Methods for Determining χ1 Angles of Aromatic Residues in Proteins from Three-Bond JC 'Cγ and J NCγ Couplings. *Journal of the American Chemical Society* **119**, 1803-1804 (1997).
9 Vögeli, B., Kazemi, S., Güntert, P. & Riek, R. Spatial elucidation of motion in proteins by ensemble-based structure calculation using exact NOEs. *Nature structural & molecular biology* **19**, 1053-1057 (2012).
10 Chi, C. N., Strotz, D., Riek, R. & Vögeli, B. Extending the eNOE data set of large proteins by evaluation of NOEs with unresolved diagonals. *Journal of biomolecular NMR* **62**, 63-69 (2015).
11 Strotz, D. *et al.* Protein allostery at atomic resolution. *Angewandte Chemie International Edition* **59**, 22132-22139 (2020).
12 Orts, J., Vögeli, B. & Riek, R. Relaxation matrix analysis of spin diffusion for the NMR structure calculation with eNOEs. *Journal of chemical theory and computation* **8**, 3483-3492 (2012).
13 Strotz, D., Orts, J., Chi, C. N., Riek, R. & Vögeli, B. ENORA2 exact NOE analysis program. *Journal of chemical theory and computation* **13**, 4336-4346 (2017).
14 Güntert, P., Mumenthaler, C. & Wüthrich, K. Torsion angle dynamics for NMR structure calculation with the new program DYANA. *Journal of molecular biology* **273**, 283-298 (1997).
15 Zhang, J. *et al.* Crystallographic and nuclear magnetic resonance evaluation of the impact of peptide binding to the second PDZ domain of protein tyrosine phosphatase 1E. *Biochemistry* **49**, 9280-9291 (2010).
16 Vögeli, B., Güntert, P. & Riek, R. Multiple-state ensemble structure determination from eNOE spectroscopy. *Molecular Physics* **111**, 437-454 (2013).
17 Fushman, D., Tjandra, N. & Cowburn, D. An approach to direct determination of protein dynamics from 15N NMR relaxation at multiple fields, independent of variable 15N chemical shift anisotropy and chemical exchange contributions. *Journal of the American Chemical Society* **121**, 8577-8582 (1999).
18 Lipari, G. & Szabo, A. Model-free approach to the interpretation of nuclear magnetic resonance relaxation in macromolecules. 1. Theory and range of validity. *Journal of the American Chemical Society* **104**, 4546-4559 (1982).
19 d'Auvergne, E. J. & Gooley, P. R. Optimisation of NMR dynamic models I. Minimisation algorithms and their performance within the model-free and Brownian rotational diffusion spaces. *Journal of biomolecular NMR* **40**, 107-119 (2008).
20 d'Auvergne, E. J. & Gooley, P. R. Set theory formulation of the model-free problem and the diffusion seeded model-free paradigm. *Molecular bioSystems* **3**, 483-494 (2007).
21 Morin, S. *et al.* relax: the analysis of biomolecular kinetics and thermodynamics using NMR relaxation dispersion data. *Bioinformatics* **30**, 2219-2220 (2014).
