# Supplementary material for: Atomic resolution protein allostery from the multi-state structure of a PDZ domain
Source: Nat Commun. 2022 Oct 20;13:6232. doi: 10.1038/s41467-022-33687-x (PMC9584909; doi:10.1038/s41467-022-33687-x)
Supplement: Supplementary file 2 — Reporting Summary [file 41467_2022_33687_MOESM2_ESM.pdf]

nature portfolio

Corresponding author(s): Beat Vögeli, Roland Riek

Last updated by author(s): 2022-08-03

Reporting Summary

Nature Portfolio wishes to improve the reproducibility of the work that we publish. This form provides structure for consistency and transparency in reporting. For further information on Nature Portfolio policies, see our Editorial Policies and the Editorial Policy Checklist.

Statistics

For all statistical analyses, confirm that the following items are present in the figure legend, table legend, main text, or Methods section.

n/a	Confirmed	
☐	☒	The exact sample size (*n*) for each experimental group/condition, given as a discrete number and unit of measurement
☒	☐	A statement on whether measurements were taken from distinct samples or whether the same sample was measured repeatedly
☒	☐	The statistical test(s) used AND whether they are one- or two-sided<br>*Only common tests should be described solely by name; describe more complex techniques in the Methods section.*
☒	☐	A description of all covariates tested
☒	☐	A description of any assumptions or corrections, such as tests of normality and adjustment for multiple comparisons
☐	☒	A full description of the statistical parameters including central tendency (e.g. means) or other basic estimates (e.g. regression coefficient) AND variation (e.g. standard deviation) or associated estimates of uncertainty (e.g. confidence intervals)
☒	☐	For null hypothesis testing, the test statistic (e.g. *F*, *t*, *r*) with confidence intervals, effect sizes, degrees of freedom and *P* value noted<br>*Give P values as exact values whenever suitable.*
☒	☐	For Bayesian analysis, information on the choice of priors and Markov chain Monte Carlo settings
☒	☐	For hierarchical and complex designs, identification of the appropriate level for tests and full reporting of outcomes
☒	☐	Estimates of effect sizes (e.g. Cohen's *d*, Pearson's *r*), indicating how they were calculated

Our web collection on statistics for biologists contains articles on many of the points above.

Software and code

Policy information about availability of computer code

Data collection	CYANA for structure calculation (version 3.98)<br>TOPSPIN for NMR spectra transformation (version 4.0)<br>NMRPipe for peak picking and intensity identification as written in the manuscript (version 10.1)
Data analysis	PDBCOR for structure analysis as written in the manuscript (version 1.0), RELAX for NMR relaxation analysis (version 4.1.3)

For manuscripts utilizing custom algorithms or software that are central to the research but not yet described in published literature, software must be made available to editors and reviewers. We strongly encourage code deposition in a community repository (e.g. GitHub). See the Nature Portfolio guidelines for submitting code & software for further information.

Data

Policy information about availability of data

All manuscripts must include a data availability statement. This statement should provide the following information, where applicable:

- Accession codes, unique identifiers, or web links for publicly available datasets
- A description of any restrictions on data availability
- For clinical datasets or third party data, please ensure that the statement adheres to our policy

The protein structure data that support the findings of this study have been deposited in the Protein Data Bank under accession codes 3LNX [https://doi.org/10.2210/pdb3LNX /pdb] (apo PDZ2) and 3LNY [https://doi.org/10.2210/pdb3LNY/pdb] (PDZ2 bound to RA-GEF2 peptide). The protein structure data that was generated in this study have been deposited in the Protein Data Bank under accession codes 7QCX [https://doi.org/10.2210/pdb7QCX/pdb] (apo PDZ2) and 7QCY [https://doi.org/10.2210/pdb7QCY/pdb] (PDZ2 bound to RA-GEF2 peptide) and in the Biological Magnetic Resonance Bank under the accession codes 34688 [https://bmrb.io/data_library/summary/index.php?bmrbId=34688] and 34689 [https://bmrb.io/data_library/summary/index.php?bmrbId=34689].

Human research participants

Policy information about studies involving human research participants and Sex and Gender in Research.

Reporting on sex and gender	NA
Population characteristics	NA
Recruitment	NA
Ethics oversight	NA

Note that full information on the approval of the study protocol must also be provided in the manuscript.

Field-specific reporting

Please select the one below that is the best fit for your research. If you are not sure, read the appropriate sections before making your selection.

☒ Life sciences ☐ Behavioural & social sciences ☐ Ecological, evolutionary & environmental sciences

For a reference copy of the document with all sections, see nature.com/documents/nr-reporting-summary-flat.pdf

Life sciences study design

All studies must disclose on these points even when the disclosure is negative.

Sample size	20 best two-state conformers (40 structural entities) were selected as in previously reported literature (Vögeli, et al., 2016)
Data exclusions	No data exclusions were performed
Replication	All experiments including PDZ structure calculations and correlation analyses were successfuly replicated multiple times
Randomization	Randomization was not applicable to this study as protein structure order does not affect the outcome of the PDBcor
Blinding	No blinding was performed as it was not applicable to this study

Reporting for specific materials, systems and methods

We require information from authors about some types of materials, experimental systems and methods used in many studies. Here, indicate whether each material, system or method listed is relevant to your study. If you are not sure if a list item applies to your research, read the appropriate section before selecting a response.

Materials & experimental systems

n/a	Involved in the study
☒	☐ Antibodies
☒	☐ Eukaryotic cell lines
☒	☐ Palaeontology and archaeology
x	☐ Animals and other organisms
☒	☐ Clinical data
☒	☐ Dual use research of concern

Methods

n/a	Involved in the study
☒	☐ ChIP-seq
☒	☐ Flow cytometry
☒	☐ MRI-based neuroimaging

Animals and other research organisms

Policy information about studies involving animals; ARRIVE guidelines recommended for reporting animal research, and Sex and Gender in Research

Laboratory animals	NA
Wild animals	NA
Reporting on sex	NA
Field-collected samples	NA
Ethics oversight	NA

Note that full information on the approval of the study protocol must also be provided in the manuscript.
